# Methylation of *MdMYB1* locus mediated by RdDM pathway regulates anthocyanin biosynthesis in apple

**DOI:** 10.1111/pbi.13337

**Published:** 2020-01-30

**Authors:** Shenghui Jiang, Nan Wang, Min Chen, Rui Zhang, Qingguo Sun, Haifeng Xu, Zongying Zhang, Yicheng Wang, Xiuqi Sui, Sufang Wang, Hongcheng Fang, Weifang Zuo, Mengyu Su, Jing Zhang, Zhangjun Fei, Xuesen Chen

**Affiliations:** ^1^ College of Horticulture Science and Engineering State Key Laboratory of Crop Biology Collaborative Innovation Center of Fruit & Vegetable Quality and Efficient Production in Shandong Shandong Agricultural University Tai'an China; ^2^ Chinese Academy of Sciences Yantai Institute of Coastal Zone Research Yantai China; ^3^ Heze University Heze China; ^4^ Yantai Modern Fruit Industry Development Company Yantai Modern Fruit Industry Research Institute Yantai China; ^5^ Shaanxi Fruit Industry Center Xi'an China; ^6^ Boyce Thompson Institute Cornell University Ithaca NY USA

**Keywords:** apple, anthocyanin, *MdMYB1* promoter, DNA methylation, AGO4, RdDM

## Abstract

Methylation at the *MdMYB1* promoter in apple sports has been reported as a regulator of the anthocyanin pathway, but little is known about how the locus is recognized by the methylation machinery to regulate anthocyanin accumulation. In this study, we analysed three differently coloured ‘Fuji’ apples and found that differences in the transcript levels of *MdMYB1*, which encodes a key regulator of anthocyanin biosynthesis, control the anthocyanin content (and therefore colour) in fruit skin. The CHH methylation levels in the MR3 region (−1246 to −780) of the *MdMYB1* promoter were found to be negatively correlated with *MdMYB1* expression. Thus, they were ideal materials to study DNA methylation in apple sports. The protein of RNA‐directed DNA methylation (RdDM) pathway responsible for CHH methylation, MdAGO4, was found to interact with the *MdMYB1* promoter. MdAGO4s can interact with MdRDM1 and MdDRM2s to form an effector complex, fulfilling CHH methylation. When *MdAGO4s* and *MdDRM2s* were overexpressed in apple calli and *Arabidopsis* mutants, those proteins increase the CHH methylation of AGO4‐binding sites. In electrophoretic mobility shift assays, MdAGO4s were found to specifically bind to sequence containing ATATCAGA. Knockdown of *MdNRPE1* did not affect the binding of MdAGO4s to the c3 region of the *MdMYB1* promoter in 35S::*AGO4* calli. Taken together, our data show that the *MdMYB1* locus is methylated through binding of MdAGO4s to the *MdMYB1* promoter to regulate anthocyanin biosynthesis by the RdDM pathway.

## Introduction

Somatic mutations, also referred to as ‘bud sports’ or simply ‘sports’, represent an important method to generate superior cultivars of woody crop species. In fruit trees, somatic mutations can alter developmental characteristics such as bearing habit, fruit‐set behaviour, fruit size, shape, colour and timing of maturity (Petit and Hampe, 2006). Sport varieties account for more than 30% of the popularized fruit varieties in China, especially those of citrus and apple (Chen *et al.*, 2015). Among fruit tree sports, fruit colour mutations are the most frequent. For example, the famous ‘Red Delicious’ apple is a sport cultivar from ‘Delicious’ and now forms a global brand (Iglesias *et al.*, 1999). Another apple cultivar, ‘Fuji’, is also very popular in China. ‘Nagafu 2’, introduced from Japan, is a sport of ‘Fuji’. Several red sports of ‘Fuji’ have been selected because of its poor coloration. The popularization of the red sports of ‘Fuji’ has promoted the rapid development of the apple industry in China. The apple cultivation in China has grown from 1133 thousand hectares in the 1990s to 2667 thousand hectares today. China is now the leading apple‐producing country and accounts for half of the cultivated area and yield of apples worldwide (Wang and Shi, 2017).

Fruit colour is determined by the composition and content of anthocyanins. Anthocyanins are water‐soluble flavonoid compounds that are responsible for colours in various plants including apple (Telias *et al.*, 2011; El‐Sharkawy *et al.*, 2015), pear (Feng *et al.*, 2010; Wang *et al.*, 2013), grape (Hichri *et al.*, 2011; Xi *et al.*, 2016) and maize (Cone *et al.*, 1993). These compounds play vital roles in seed dispersal (Winkelshirley, 2001), plant resistance (Field *et al.*, 2001) and protection against ultraviolet radiation (Schaefer *et al.*, 2004), and are beneficial for human health to protect against diseases (Liu *et al.*, 2005; Rossi *et al.*, 2003; Tsuda *et al.*, 2003). Anthocyanins are synthesized through the flavonoid pathway, which includes both structural and regulatory genes. The structural genes can be divided into two groups: those encoding enzymes that catalyse the early steps of the pathway including *CHS* (chalcone synthase), *CHI* (chalcone isomerase) and *F3H* (flavanone 3‐hydroxylase) to produce colourless dihydroflavonol compounds; and those encoding enzymes that catalyse the later steps of the pathway, including *DFR* (dihydroflavonol 4‐reductase), *ANS* (anthocyanidin synthase) and *UFGT* (UDP‐glucose: flavonoid 3‐O‐glucosyltransferase) to produce coloured anthocyanins (Baudry *et al.*, 2004). Three transcription factors, R2R3‐MYB, bHLH (basic helix–loop–helix) and TTG1 (WD40 repeat‐containing protein), form the MBW (MYB‐bHLH‐WD40) complex, which regulates anthocyanin biosynthesis at the transcriptional level (Gonzalez *et al.*, 2008; Petroni and Tonelli, 2011; Xu *et al.*, 2015).

Differences in anthocyanin contents between a bud sport and its parent are reported to be due to the diverse methylation of *MYB1/10* in fruit tissues. For example, in a red bud sport, the methylation level of the *MdMYB1* promoter was found to be lower than that in ‘Ralls’ (Xu *et al.*, 2012). In the anthocyanin‐deficient yellow‐skinned apple mutant ‘Blondee’, the methylation level of the *MdMYB10* promoter was higher than that in its parent ‘Kidd's D‐8’ (El‐Sharkawy *et al.*, 2015). Methylation of the *PcMYB10* promoter was shown to be responsible for the green skin of the bud sport of ‘Max Red Bartlett’ pear (Wang *et al.*, 2013). Therefore, apple mutants are excellent materials for research on the DNA methylation in fruit trees.

In plants, DNA methylation occurs in three different sequence contexts: CG, CHG and CHH (H = A, T or C). In *Arabidopsis thaliana*, a methyl group is transferred to the cytosine bases of DNA to form 5‐methylcytosine by four types of DNA methyltransferases: methyltransferase1 (MET1) (Ronemus *et al.*, 1996), chromomethylase3 (CMT3) (Lindroth and Jacobsen, 2001), domains rearranged methyltransferase2 (DRM2) (Cao and Jacobsen, 2002) and CMT2 (Stroud *et al.*, 2014). The maintenance of CG and CHG methylation is mainly controlled by MET1 and CMT3, while CHH methylation is controlled by DRM2. In apple fruit, different CHH methylation levels at *MdMYB10* loci were detected between the yellow‐skinned somatic mutant ‘Blondee’ and its red‐skinned parent ‘Kidd's D‐8’ (El‐Sharkawy *et al.*, 2015). An analysis of apple leaf and fruit methylomes demonstrated that higher CHH DNA methylation levels were globally exhibited in fruit (Daccord *et al.*, 2017). Those findings suggested that RNA‐directed DNA methylation (RdDM) may play an essential role in apple fruit. RdDM is an important process in repressive epigenetic regulation that can initiate transcriptional gene silencing (TGS) (Matzke and Mosher, 2014; Wassenegger *et al.*, 1994). There are many key components of the RdDM pathway, such as Argonaute 4 (AGO4) (Li *et al.*, 2006; Ye *et al.*, 2012), RNA‐directed DNA methylation1 (RDM1) (Gao *et al.*, 2010) and DRM2 (Zhong *et al.*, 2014). The non‐coding RNAs involved in targeting AGO4 to specific genomic loci are probably long non‐coding RNAs (lncRNAs) produced by plant‐specific RNA polymerase V (PolV) (Wierzbicki *et al.*, 2008). The unique activity of PolV is attributed primarily to its largest subunits, NRPE1 (El‐Shami et al., 2007). The binding of AGO4 to gene promoters recruits DRM2, which catalyses CHH methylation (Zheng *et al.*, 2012).

To elucidate the mechanism of different methylation at the *MdMYB1* locus, we characterized the red sports ‘Yanfu 3’ and ‘Yanfu 8’ and their parents ‘Nagafu 2’ and ‘Yanfu 3’, respectively, all of which are varieties of ‘Fuji’ apple. The expression patterns of *MdMYB1* were directly correlated with anthocyanin contents in the three cultivars, and were negatively correlated with the CHH methylation level of the *MdMYB1* promoter. We analysed the relationship between three kinds of proteins (MdAGO4s, MdDRM2s and MdRDM1) in the RdDM pathway and *MdMYB1*, and found that MdAGO4s could interact with the *MdMYB1* promoter in vivo and in vitro. The three kinds of proteins (AGOs, DRM2s and RMD1) in apple were able to interact with each other and form an effector complex. Overexpression of *MdAGO4‐1/2* and *MdDRM2‐1/2* in apple callus increased the CHH methylation level of the *MdMYB1* promoter, suggesting that the RdDM pathway could modify methylation of the *MdMYB1* locus, to further affect the anthocyanin biosynthesis in apple. Overexpression of *MdAGO4‐1/2* and *MdDRM2‐1/2* in *Arabidopsis* mutants was able to rescue the CHH methylation level at AGO4‐binding sites. Interestingly, electrophoretic mobility shift assays (EMSAs) demonstrated that MdAGO4s could specifically bind to the sequence ATATCAGA. The binding of MdAGO4s was not affected by knockdown of MdNRPE1 in *MdAGO4‐1/2*‐overexpressing callus. Together, these findings revealed that *MdMYB1* methylation can be modified by the RdDM pathway in apple fruit and that MdAGO4s bind directly to DNA at the ATATCAGA sequence. Those findings provide new insights into the functions of the RdDM pathway in apple.

## Results

### Anthocyanin accumulation and relative gene expression in apples after bag removal

**Figure 1:**
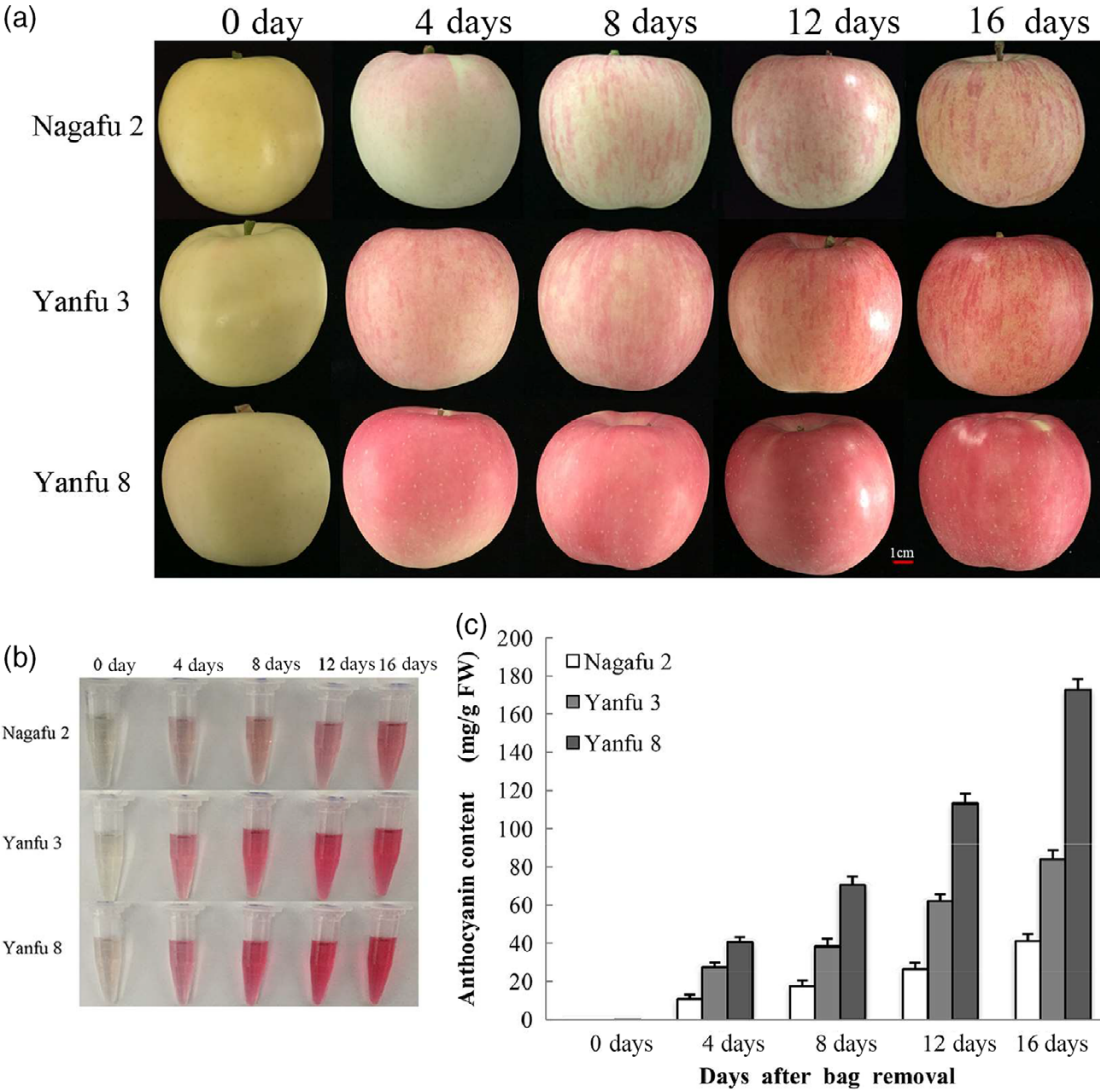
Fruit development and anthocyanin accumulation after bag removal. (a) Fruit development of ‘Nagafu 2’, ‘Yanfu 3’ and ‘Yanfu 8’ at different days after bag removal. (b) Anthocyanin accumulation in three apple varieties after bag removal. (c) Anthocyanin contents in fruit skins of three apple varieties after bag removal.

Anthocyanin accumulation in ‘Nagafu 2’, ‘Yanfu 3’ and ‘Yanfu 8’ fruit skin was monitored after bag removal (Figure 1a,b). In ‘Nagafu 2’ fruit skin, anthocyanin accumulated gradually from 4 to 16 days after bag removal (DABR), peaked at 16 DABR (Figure 1c) and formed a red‐striped pattern. However, anthocyanin accumulated more rapidly, and to significantly higher levels, in the fruit skin of ‘Yanfu 3’ and ‘Yanfu 8’ (Figure 1c). The fruit skin of ‘Yanfu 8’ had the highest anthocyanin concentration and a fully red pattern, different from the red‐striped pattern in ‘Nagafu 2’ and ‘Yanfu 3’.

The structural genes in anthocyanin biosynthesis in apple include *MdCHS*, *MdCHI*, *MdF3H*, *MdDFR*, *MdANS* and *MdUFGT*. The transcript profiles of these genes were investigated in fruit skins of ‘Nagafu 2’, ‘Yanfu 3’ and ‘Yanfu 8’ (Figure S1a). The transcript levels of all these genes were higher in ‘Yanfu 3’ and ‘Yanfu 8’ than in ‘Nagafu 2’ at each time point (Figure S1b). Their transcript levels peaked at 12 DABR in ‘Yanfu 3’ and ‘Yanfu 8’, while those of *MdCHS*, *MdANS* and *MdUFGT* peaked at the last time point in ‘Nagafu 2’.

We also investigated the transcription profiles of three regulatory genes in the three cultivars (Figure S1b). The transcript level of *MdMYB1* was highest in ‘Yanfu 8’. The transcription profiles of *MdMYB1* were very similar to those of the structural genes (*MdCHI*, *MdF3H*, *MdDFR*, *MdANS* and *MdUFGT*). In all three cultivars, the highest transcript levels of *MdMYB1* were detected at 12 DABR. The transcript levels of *MdbHLH3* and *MdbHLH33* were similar to those of *MdMYB1*, with the highest transcript levels also detected at 12 DABR (Figure S1b). Among the three transcription factors, *MdMYB1* showed the largest increases in transcript levels after bag removal, with moderate increases in the paler cultivar ‘Nagafu 2’ and large increases in the deep‐red cultivars ‘Yanfu 3’ and ‘Yanfu 8’. Among the three transcription factors, *MdMYB1* was predominately expressed. These results indicated that the higher expression levels of *MdMYB1* might up‐regulate expression of anthocyanin structural genes in ‘Yanfu 3’ and ‘Yanfu 8’.

### Genetic and epigenetic characterization of *MdMYB1*

To determine why *MdMYB1* was expressed at higher levels in ‘Yanfu 3’ and ‘Yanfu 8’ than in ‘Nagafu 2’, the cDNA coding sequences and genomic DNA (gDNA) sequences of *MdMYB1* were isolated from fruit skins at all three cultivars at 12 DABR based on the sequence of *MdMYB1‐1* (DQ886414). The gDNA included a promoter region of 1,657 bases upstream from the translation start site of *MdMYB1*. Sequence analyses showed that there were no base mutations in the cDNA or gDNA sequences among the three cultivars. This result demonstrated that genetic mutation was not the reason for the different coloration of the three apple cultivars.

**Figure 2:**
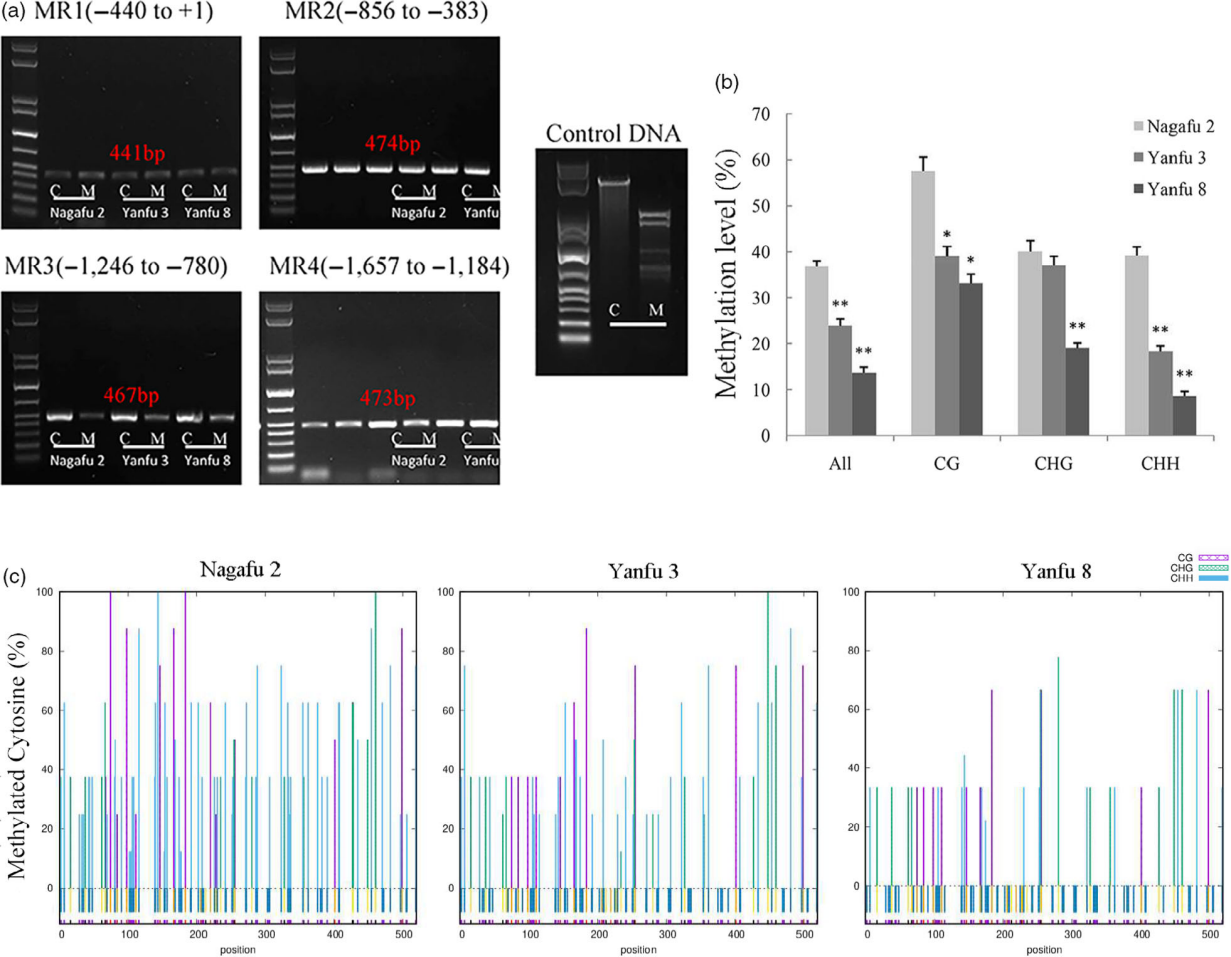
Regions in *MdMYB1* promoter with different methylation levels among ‘Nagafu 2’, ‘Yanfu 3’ and ‘Yanfu 8’. (a) Methylation levels at different promoter regions of *MdMYB1* in ‘Nagafu 2’, ‘Yanfu 3’ and ‘Yanfu 8’ determined by McrBC‐PCR with genomic DNA from fruit skin samples (12 DABR) as the template. Promoter of *MdMYB1* was divided into four regions (MR1–MR4; location of each region is marked). Negative control template contained water instead of GTP. M and C represent McrBC‐digestion groups with or without GTP, respectively. Plasmid DNA was used as positive control. (b) Methylation levels of MR3 promoter region of *MdMYB1* determined by BSP. On x‐axis, ‘All’ represents overall methylated cytosine, while CG, CHG and CHH represent three different types of cytosine methylation. (c) Average percentage of methylation at each cytosine in three cultivars. Error bars show standard deviation of data from triplicate *g*DNA extractions with three technical replicates, ‘*’ and ‘**’ indicate significance difference at *P* < 0.05 and *P* < 0.01, respectively.

Next, we investigated the methylation level of the *MdMYB1* promoter region. The McrBC analyses indicated that only one region, MR3 (−1,246 to −780), showed differences in methylation levels among the three cultivars (Figure 2a). The MR1 (−440 to +1) and MR2 (−856 to −383) regions exhibited low methylation levels in all three cultivars. The MR4 region (−1657 to −1184) showed high methylation levels only in ‘Yanfu 3’ (Figure 2a). Thus, the promoter region MR3 (−1246 to −780) that showed visible differences in methylation levels among the three cultivars was selected for further analyses.

To validate these results, the methylation levels of the MR3 region of the *MdMYB1* promoter in ‘Nagafu 2’, ‘Yanfu 3’ and ‘Yanfu 8’ were further analysed by BS‐PCR. Analyses of sequence data indicated that the MR3 region displayed diverse methylation levels among the three cultivars (Figure 2b,c). The overall methylation level of the MR3 region was highest in ‘Nagafu 2’, lower in ‘Yanfu 3’ and lowest in ‘Yanfu 8’. The pattern of CHH methylation (where H is A, C or T) was very similar to the overall methylation pattern in the three cultivars. The CG methylation was similar in ‘Yanfu 3’ and ‘Yanfu 8’, and much higher in ‘Nagafu 2’. The CHG methylation was almost the same in ‘Nagafu 2’ and ‘Yanfu 3’ but lower in ‘Yanfu 8’. The CHH methylation was more abundant than CG and CHG methylation in the MR3 promoter region.

Correlation analyses were conducted to determine whether the differences in *MdMYB1* promoter methylation levels were associated with the differences in *MdMYB1* transcription and anthocyanin accumulation among the three cultivars. The CHH methylation of the MR3 regions showed a significant negative correlation with anthocyanin accumulation (*r*MR3 = −0.828, *P* = 1.05 × 10^−3^) and with the transcript level of *MdMYB1* (*r*MR3 = −0.876, *P* = 2.74 × 10^−4^). These results indicated that the different CHH methylation levels of *MdMYB1* promoter may affect its transcription.

Next, we identified 14 putative AGO genes from the apple genome using bioinformatics methods. A phylogenetic tree analysis between apple and *Arabidopsis* AGO revealed two AGO genes, *MdAGO4‐1* and *MdAGO4‐2*, homologous to *AtAGO4* (Figure S2); two identified two DRM2 genes, *MdDRM2‐1* and *MdDRM2‐2*, homologous to *AtDRM2* (Figure S3); and an *AtRMD1* homolog, *MdRDM1*. We analysed the transcription patterns of *MdAGO4s*, *MdDRM2*s and *MdRDM1*. The transcript levels of *MdAGO4s* and *MdRDM1* were no particular pattern, while those of the two *MdDRM2s* were lower in the sports than in their parents during apple coloration (Figure S1). We considered that the lower transcript levels of *MdDRM2s* may be involved in maintaining the CHH methylation of genes.

### Binding of MdAGO4‐1/2 to the *MdMYB1* promoter

**Figure 3:**
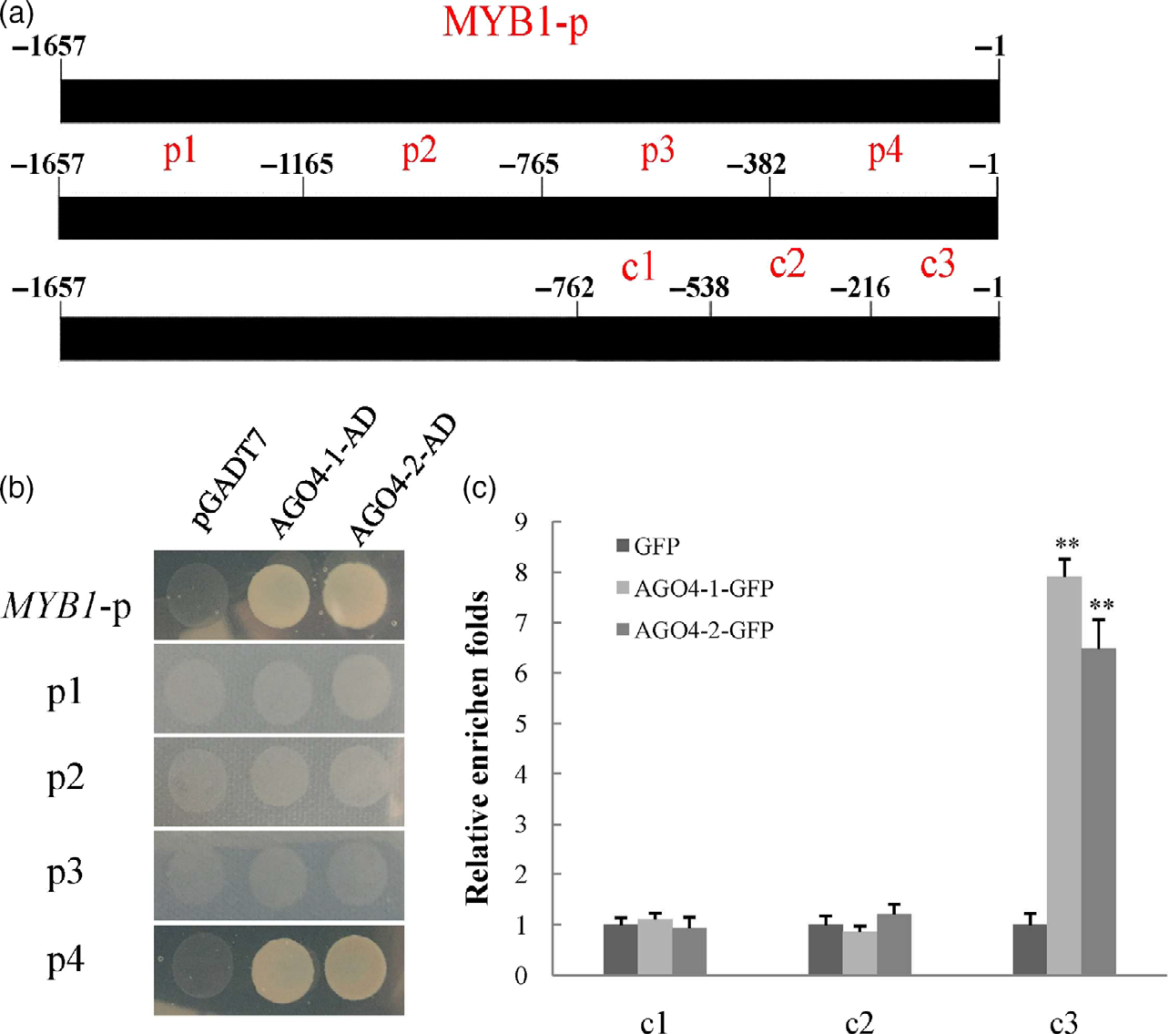
Interaction between MdAGO4‐1/2 and promoter of *MdMYB1*. (a) Regions of *MdMYB1* promoter used in interaction analyses. Black rectangles represent *MdMYB1* promoter (−1,657 to −1 bp). Four different regions (p1–4) in the *MdMYB1* promoter were used for the Y1H assays; three regions (c1–3) were designed for ChIP‐qPCR assay. (b) Y1H assays showing interactions between MdAGO4‐1/2 with the *MdMYB1* promoter and the p4 region. Empty AD vector was used as the negative control. (c) ChIP‐PCR results showing binding of MdAGO4‐1/2 to c3 region of *MdMYB1* promoter *in vivo*. Apple ‘Orin’ callus overexpressing GFP protein was used as the control. Error bars show standard derivation of three replicates, ‘**’ indicates significance at *P* < 0.01.

In *Arabidopsis*, AGO4 binds to promoter regions and directs CHH methylation, which controls the expression of target genes (Zheng *et al.*, 2012). We found that differences in CHH methylation might regulate *MdMYB1* transcription; thus, it is possible that MdAGO4 binds to the *MdMYB1* promoter. To investigate whether MdAGO4s bind to the *MdMYB1* promoter (Figure 3a), yeast one‐hybrid (Y1H) assays were performed. In the Y1H assays, MdAGO4‐1/2 bound to the *MdMYB1* promoter (Figure 3b). To identify the exact region in the *MdMYB1* promoter that MdAGO4‐1/2 bind to, four fragments (p1–p4) of the *MdMYB1* promoter were separately cloned into the pHIS2 vector (Figure 3a). In Y1H assays, MdAGO4‐1/2 only bound to the p4 fragment (−382 to −1) (Figure 3b).

To validate these results *in vivo*, ChIP‐qPCR assays were conducted using the 35S::*AGO4‐1*‐GFP, 35S::*AGO4‐2*‐GFP and 35S::GFP transgenic apple calli. The −538 to −1 region was divided into two fragments, c2 and c3; the c1 fragment was used as the control as it was verified to not bind to MdAGO4‐l/2 in Y1H assays. The c3 region of *MYB1*‐p4 was enriched in the 35S::*AGO4‐1*‐GFP and 35S::*AGO4‐2*‐GFP transgenic calli compared with the 35S::GFP control (Figure 3c), indicating that MdAGO4‐1/2 was able to bind to *MdMYB1* promoter (−216 to −1) *in vivo*.

### Formation of MdAGO4, MdDRM2 and MdRDM1 protein complex

In *Arabidopsis*, AGO4, DRM2 and RDM1 form a protein complex (Gao *et al.*, 2010). To test whether this interaction also occurs in apple, Co‐IP assays were performed to test the interactions among MdAGO4s, MdDRM2s and MdRMD1. In Co‐IP assays, MdAGO4‐1‐HA was immunoprecipitated by MdDRM2‐1‐FLAG, MdDRM2‐2 and MdRDM1‐FLAG in calli, indicating that MdAGO4‐l can interact with MdDRM2‐1/2 and MdRDM1; MdAGO4‐2‐HA was immunoprecipitated by MdDRM2‐1‐FLAG, MdDRM2‐2 and MdRDM1‐FLAG in calli, showing that MdAGO4‐2 can interact with MdDRM2‐1/2 and MdRDM1 (Figure S4). We also detected an interaction between MdDRM2‐1/2 and MdRDM1; results showed that MdDRM2‐1 and MdDRM2‐2 interacted with MdRDM1 (Figure S4). Pull‐down assays confirmed that the three kinds of proteins were able to interact with each other *in vitro* (Figure S5). Together, these results confirmed that the three kinds of proteins can form a protein complex.

### Heterologous expression of *MdAGO4s* and *MdDRM2s* increases CHH methylation in *Arabidopsis* mutants

**Figure 4:**
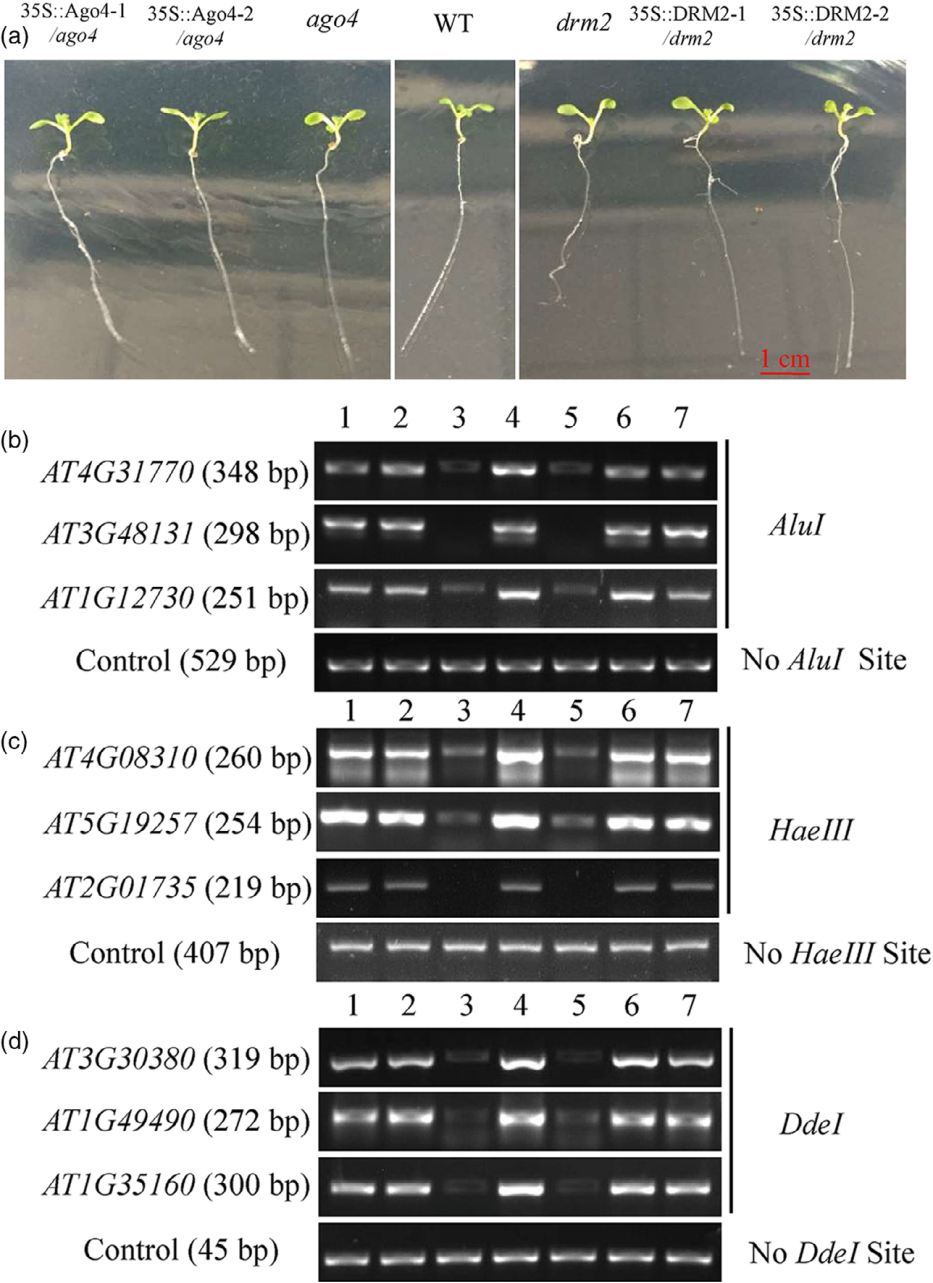
Heterologous expression of *MdAGO4s* or *MdDRM2s* rescues CHH methylation of AGO4‐binding sites in *Arabidopsis ago4* and *drm2* mutants. (a) Ten‐day‐old wild‐type *Arabidopsis* (WT), *ago4* mutant, *drm2* mutant and transgenic *Arabidopsis* (35S::*AGO4‐1/2* and *35S::DRM2‐1/2*). DNA methylation was analysed using three methylation‐sensitive restriction endonucleases, *AluI* (b), *HaeIII* (c) and *DdeI* (d). Genomic DNA was extracted from 2‐week‐old seedlings, and digested genomic DNAs were amplified by PCR. Sequences lacking *AluI* sites (IGN5), *HaeIII* (AT5G27860) and *DdeI* (AT2G36490) were used as loading controls. Sequence and size of *Arabidopsis* genes are listed in Table S2. 1–7 indicate 35S::*AGO4‐1*/*ago4*, 35S::*AGO4‐2*/*ago4*, *ago4*, WT, *drm2*, 35S::*DRM2‐1*/*drm2* and 35S::*DRM2‐2*/*drm2*, respectively.

AGO4 is required for CHH methylation of DNA at its binding sites (Zheng *et al.*, 2012). Therefore, *MdAGO4s* and *MdDRM2s* were heterologously expressed in the *Arabidopsis* mutant *ago4* and *drm2* by transformation of 35S::*AGO4‐1/2*‐GFP and 35S::*DRM2‐1/2*‐GFP fusion plasmids, respectively. We probed DNA methylation levels at 12 AGO4‐binding promoter regions in wild‐type (WT), two mutants and in the 35S::*AGO4‐1/2* and 35S::*DRM2‐1/2* transgenic lines using DNA extracted from 2‐week‐old lines (Figure 4a). Digestion with three methylation‐sensitive restriction endonucleases (*AluI*, *HaeIII*, *DdeI*) followed by PCR demonstrated that CHH methylation in AGO4‐binding promoter regions was rescued in the 35S::*AGO4‐1/2* and 35S::*DRM2‐1/2* transgenic lines (Figure 4b–d). These results showed that *MdAGO4s* and *MdDRM2s* were able to complement the deficient phenotype of the two *ago4* and *drm2* mutants and promote CHH methylation at AGO4‐binding regions.

### Overexpression of *MdAGO4s* and *MdDRM2s *increases CHH methylation of *MdMYB1* promoter in apple callus

To further elucidate the functional roles of *MdAGO4s* and *MdDRM2s*, they were overexpressed in ‘Orin’ apple calli (Figure S6a). The presence of the transgenes in calli was confirmed by Western blotting (Figure S6b). The transcript levels of anthocyanin pathway genes were investigated in ‘Orin’ wild‐type (Orin‐WT), 35S::*AGO4‐1/2* and 35S::*DRM2‐1/2* calli (Figure S7a). The transcript levels of anthocyanin structural genes were much lower in 35S::*AGO4‐1/2* and 35S::*DRM2‐1/2* calli than in Orin‐WT callus. The transcript level of *MdMYB1* was also lower in the transgenic calli than in Orin‐WT callus. The transcript levels of *MdAGO4‐1/2*, *MdDRM2‐1/2* and *MdRDM1* were higher in transgenic calli than in Orin‐WT callus (Figure S8a). The CHH methylation levels of the *MdMYB1* promoter (−1657 to −1) were 49.0% and 42.3% higher in 35S::*AGO4‐1* and 35S::*AGO4‐2* calli, respectively, than in Orin‐WT calli, and CHH methylation levels of the *MdMYB1* promoter (−1657 to −1) were 71.3% and 53.4% higher in 35S::*DRM2‐1* and 35S::*DRM2‐2* calli, respectively, than in Orin‐WT calli (Figures S6c and S9).

To confirm the functional roles of *MdAGO4s* and *MdDRM2s* in the anthocyanin pathway, they were also overexpressed in red‐fleshed apple callus (Red‐WT) (Ji *et al.*, 2015). Overexpression of *MdAGO4s* and *MdDRM2s* resulted in a pale‐red flesh colour (Figure S10a). The presence of the transgenes in calli was confirmed by Western blotting (Figure S10b). In addition, the transcript levels of anthocyanin structural and regulatory genes were significantly lower in the transgenic calli than in Red‐WT callus (Figures S7b and S8b). The CHH methylation levels of the *MdMYB10* (Espley *et al.*, 2009; Wang *et al.*, 2017) promoter (−1758 to −1) were 3.6‐fold and 3.8‐fold higher in the two *MdAGO4* transgenic calli than in Red‐WT calli, and the CHH methylation levels of the *MdMYB10* were 4.1‐fold and 3.9‐fold higher in the two *MdDRM2s* transgenic calli than in Red‐WT calli (Figures S10c and S11). These results indicated that MdAGO4s and MdDRM2s function in CHH methylation of the *MdMYB1* promoter in apple.

### MdAGO4s bind to the CHH methylated region

**Figure 5:**
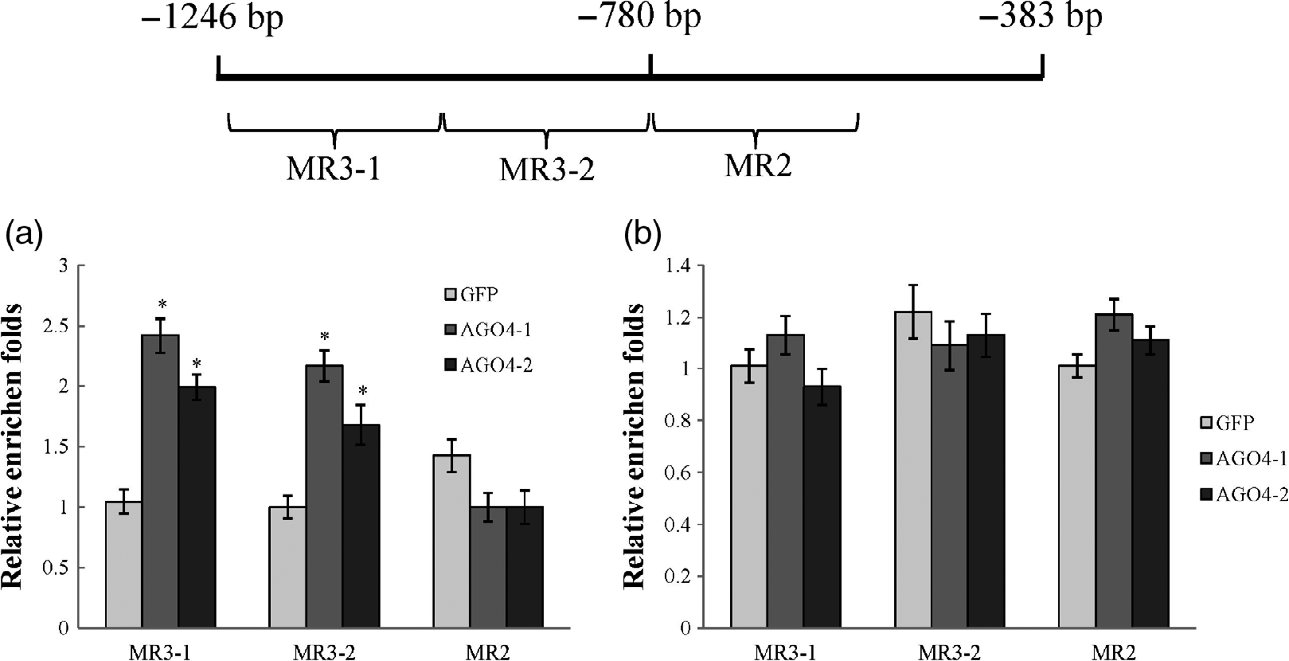
MdAGO4s bind to MR3 region. (a) ChIP‐qPCR results showing binding of MdAGO4‐1/2 to MR3 region. (b) ChIP‐qPCR results showing that binding of MdAGO4‐1/2 to MR3 region is inhibited by NRPE1 knockdown (by RNA interference). Error bars show standard derivation of three replicates. ‘*’ indicates significance at *P* < 0.05.

The binding of AGO4 to specific gene promoters is mediated by lncRNAs produced by PolⅤ (Wierzbicki *et al.*, 2009). The lncRNAs possibly involved in binding to the *MdMYB1* were identified from data published by Yang *et al.* (2019) and are listed in Table S3. Those lncRNAs were differently expressed between 0d and 8d under light. In this study, the MdAGO4s were able to bind to the *MdMYB1* promoter in Y1H, which demonstrated binding *in vitro*. The binding appeared to be direct binding. To confirm this, we performed further experience to check whether MdAGO4s bind to the CHH methylated region in apple. The results showed that MdAGO4s were able to bind to the MR3 region (Figure 5a), like in *Arabidopsis*. When we knocked down MdNRPE1 (Figure S12a,b), the binding to the MR3 region was inhibited (Figure 5b). These results indicated that the binding of MdAGO4s to the CHH methylated region is dependent on the lncRNAs. Together, these results showed that lncRNA‐mediated AGO4 binding to gene promoters is conserved in apple.

AGO4 contributes to siRNA production through its Piwi domain, which has endonuclease activity (Qi *et al.*, 2006). Individual 24‐nt siRNAs were quantitatively detected by RT‐PCR according to the method reported by Zhang *et al *(2014). We observed increased siRNA levels in the MdAGO4‐overexpressing calli (Figure S12c). Together, these results showed that MdAGO4s guided DNA methylation and are involved in siRNA production at their target loci.

### MdAGO4s directly bind to *MdMYB1 *promoter through Piwi domain *in vitro*

**Figure 6:**
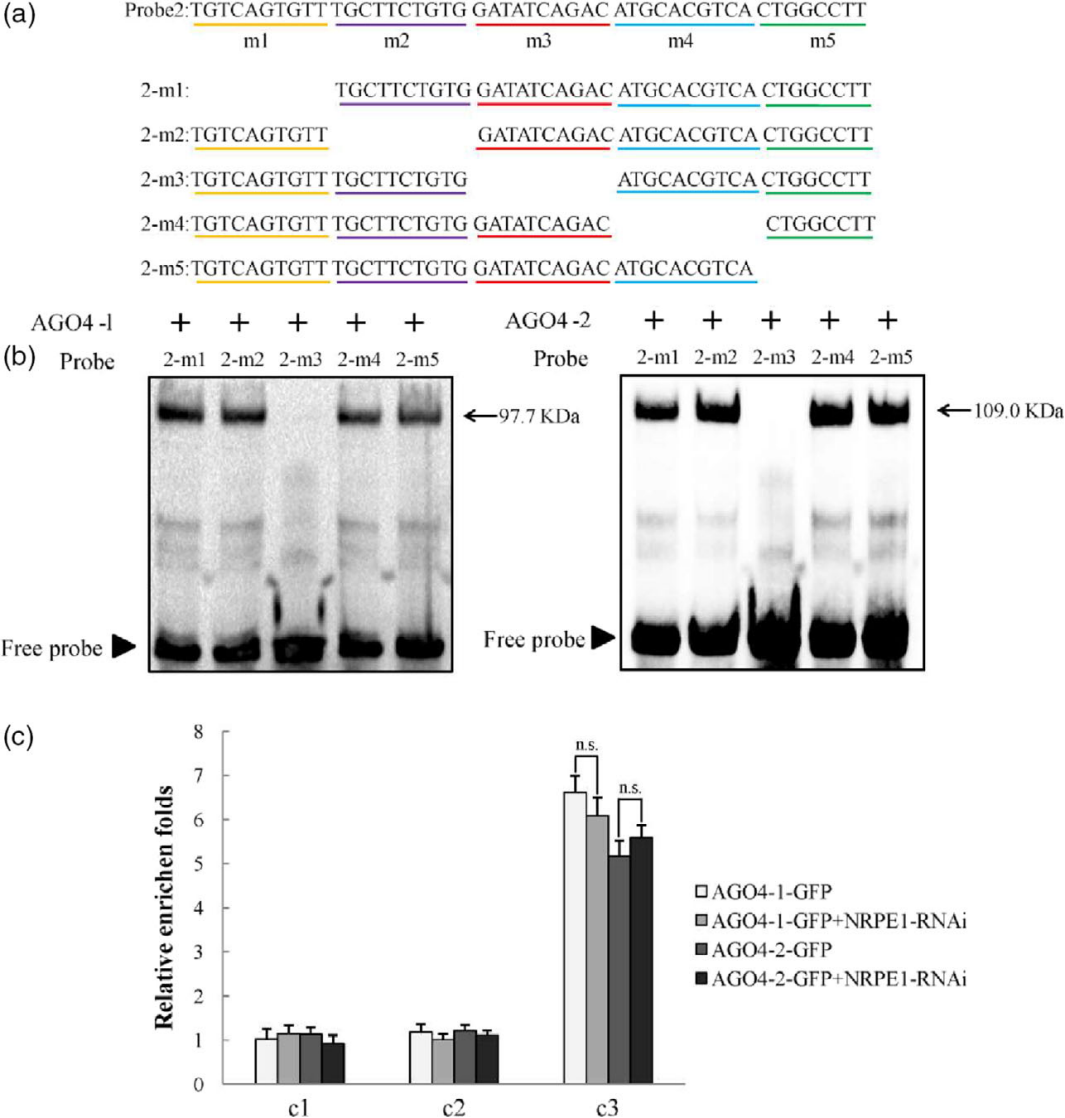
MdAGO4s directly bind to ATATCAGA sequence. (a) Five mutated probes of the probe2 used for EMSAs, 2‐m1 represents probe2 without m1 region. (b) EMSAs showing binding of MdAGO4‐1/2 to different mutated probes of probe2. (c) ChIP‐qPCR results showing binding of MdAGO4‐1/2 to c3 region without guidance by siRNA. Error bars show standard deviation of three replicates. ‘n.s.’ indicates significance at *P >* 0.01.

A previous study provided evidence that the AGO4‐DNA interaction is dependent on PolV manner (Lahmy *et al.*, 2016). Therefore, we performed EMSA to examine whether the MdAGO4s could bind to the *MdMYB1* promoter. Four probes were designed from the c3 fragment (Figure S13a). In the EMSAs, MdAGO4‐1/2 only bound to probe2 and did not bind to probe1, probe3 or probe4 (Figure S13b,c). Furthermore, five mutant probes of probe2 were designed to identify the exact region for MdAGO4‐1/2 binding (Figure 6a). In the EMSAs, both MdAGO4‐1 and MdAGO4‐2 bound to 2‐m1, 2‐m2, 2‐m4 and 2‐m5, but not to 2‐m3 (Figure 6b). In other words, an intact m3 region (GATATCAGAC) was found to be essential for MdAGO4 binding.

To verify these results, two additional probes were synthesized: an *MdFLS* (flavonol synthase) (Wang *et al.*, 2017) probe with the sequence ATATCAGA and an *MdGST* (glutathione S‐transferase) (Jiang *et al.*, 2019) probe with the sequence GATATCA (Figure S14a). In the EMSAs, MdAGO4‐1/2 bound to the *MdFLS* probe, even though this probe was shorter than the GATATCAGAC sequence (Figure S14b). The specific binding analysis indicated that MdAGO4‐1/2 specifically binds to the sequence ATATCAGA (Figure S15).

In *Arabidopsis*, the binding of AGO4 to gene promoters is mediated by lncRNAs produced by PolV. The lncRNA‐mediated AGO4 binding to gene promoters directs asymmetric DNA methylation to these regions, which regulates the gene expression of targeted genes (Wierzbicki *et al.*, 2009; Wierzbicki, 2012). Here, to eliminate the influence of lncRNAs, we knocked down *MdNRPE1* (encoding the largest subunit of PolV) in 35S::*AGO4‐1*‐GFP and 35S::*AGO4‐2*‐GFP transgenic apple calli using RNA interference (RNAi) (Figure S12a,b). In ChIP‐qPCR analyses of the two co‐transgenic apple calli, there was no significant difference in binding at the c3 region between the 35S::*AGO4‐1/2*‐GFP calli and the co‐transgenic calli (Figure 6c). Taken together, these results suggested that MdAGO4s directly bind to the ATATCAGA sequence in the c3 region of *MdMYB1* promoter.

Finally, we conducted mutation experiments to determine which domain of MdAGO4s specifically binds to the promoter of *MdMYB1*. Our analyses of MdAGO4s indicated that they contain three domains: Argonaute N domain, PAZ domain and Piwi domain (Figure S16a). We generated the MdAGO4 mutants, one with a deleted PAZ domain and the other with a deleted Piwi domain (Figure S16a). In EMSAs, MdAGO4s without the Piwi domain could not bind to the probe, while MdAGO4s without the PAZ domain could (Figure S16b,c). The results indicated that the Piwi domain is the main site where MdAGO4s bind to DNA.

## Discussion

### Differences in coloration patterns among apple varieties result from differences in transcript levels of anthocyanin biosynthetic genes

To understand the mechanism of the mutation in the three differently coloured ‘Fuji’ apple varieties (‘Nagafu 2’, ‘Yanfu 3’ and ‘Yanfu 8’), the transcript levels of anthocyanin structural and regulatory genes in fruit skin were monitored after removing bags from ripening fruits. The transcript levels of anthocyanin biosynthetic genes were higher in the deeply pigmented cultivars ‘Yanfu 3’ and ‘Yanfu 8’ than in the pale‐skinned ‘Nagafu 2’ at each sampled time point (Figure S1b). These results are similar to those reported for ‘Cripps’ Red and ‘Mutsu’ apple (Bai *et al.*, 2016; Takos *et al.*, 2006) and other fruits including pear (Feng *et al.*, 2010; Wang *et al.*, 2013), peach (Tuan *et al.*, 2015) and sweet cherry (Jin *et al.*, 2016).

Anthocyanins are synthesized via a branch of the flavonoid pathway. Previous studies have isolated and characterized structural anthocyanin biosynthetic genes including *CHS*, *CHI*, *F3H*, *DFR*, *ANS* and *UFGT* (Jaakola, 2013). These genes encode the enzymes in the later part of the anthocyanin biosynthetic pathway, and their transcription is controlled by the MBW protein complex (Xu *et al.*, 2015). The key component of the MBW complex in apple is encoded by *MdMYB1*, which is allelic to *MdMYBA* (Ban *et al.*, 2007) and *MdMYB10* (Espley *et al.*, 2007). All these *MYB* alleles are located at the same position in linkage group 9 (Chagné *et al.*, 2007; Linwang *et al.*, 2010). In this study, we found that the transcript levels of *MdMYB1* and *MdbHLH3* and *MdbHLH33* were up‐regulated in the red‐skinned apples. We considered that this is because these two bHLH transcription factors interact with MdMYB1 to form a protein complex. Consistently, the transcript levels of *MdbHLH3* and *MdbHLH33* were also higher in ‘Yanfu 3’ and ‘Yanfu 8’. Thus, *MdMYB1* was identified as the master regulatory factor causing differences in coloration among these three apple varieties.

### Epigenetic regulation of *MdMYB1* affects anthocyanin biosynthesis in apple sports

Mutations in regulatory genes of anthocyanin biosynthesis that alter the abundance of transcription factors have been reported. For example, in white grape (*V. vinifera*), a retrotransposon‐induced mutation in *VvmybA1* promoter resulted in the decreased level of *VvmybA1* transcripts, which further led to an anthocyanin‐reduced phenotype (Kobayashi *et al.*, 2004). A Copia‐like retrotransposon was inserted into the MYB transcriptional activator of anthocyanin production in Sicilian blood orange, *Ruby*, and controlled its expression (Butelli *et al.*, 2012). In red‐fleshed apple, an insertion of a minisatellite in the promoter of *MdMYB10* was responsible for the increased anthocyanin content (Espley *et al.*, 2009). In this study, the transcript level of *MdMYB1* was lower in the parents than in their red sports. To explore the mechanism underlying the differences in transcript levels of *MdMYB1*, we cloned the mRNA and regulatory region of *MdMYB1* from all three cultivars. No sequence differences were detected, indicating that the differences in the transcript levels of *MdMYB1* among the three cultivars might result from differences in the methylation of its promoter region.

To test this hypothesis, we analysed the methylation of the *MdMYB1* promoter using an McrBC‐PCR approach. Our analyses indicated the difference in methylation levels among the three cultivars at the promoter region of *MdMYB1* from −1246 to −780 bp of the translation start site (Figure 2a, MR3 region). Further analysis using BSP indicated that the overall methylation level of the MR3 region was lower in ‘Yanfu 3’ and ‘Yanfu 8’ than in ‘Nagafu 2’. These results are similar to those obtained in analyses of apple varieties Honey Crisp with a striped phenotype (Telias *et al.*, 2011) and ‘Ralls’ with a red spot phenotype (Xu *et al.*, 2012). To better understand the differences in methylation among ‘Nagafu 2’, ‘Yanfu 3’ and ‘Yanfu 8’, we also analysed the three types of cytosine methylation (CHH, CHG and CG) in the MR3 region. The CHH cytosine methylation patterns were very similar to the overall methylation patterns in the three cultivars (Figure 2b), and CHH cytosine methylation was significantly negatively correlated with anthocyanin accumulation and with the transcript level of *MdMYB1*. Similar differences in methylation patterns were detected between the yellow‐skinned somatic mutant ‘Blondee’ and its red‐skinned parent ‘Kidd's D‐8’ (El‐Sharkawy *et al.*, 2015). These data revealed that, among the three types of cytosine methylation, CHH methylation showed significant differences among the three cultivars. This result was also consistent with those reported in a recent study on genome‐wide DNA methylation dynamics in apple, which suggested that RNA‐directed DNA methylation may play an essential role in fruit (Daccord *et al.*, 2017). Thus, these apple mutants are ideal materials for studying DNA methylation and will provide some useful information for apple sport breeding.

### RdDM landscapes CHH methylation

Unlike in mammals (Law and Jacobsen, 2010), plants show DNA methylation in three different sequence contexts: CG, CHG and CHH. MET1 catalyses CG methylation (Saze *et al.*, 2003), CMT3 catalyses CHG methylation (Lindroth and Jacobsen, 2001), and DRM2 is responsible for CHH methylation (Cao and Jacobsen, 2002). As a *de novo* DNA methyltransferase, DRM2 is expected to be a component of the RdDM effector complex. A previous study identified an association and co‐localization between DRM2 and RDM1, which also interacts with AGO4, thus suggesting that DRM2 is indeed part of the effector complex in *Arabidopsis* (Gao *et al.*, 2010). In this study, we found that apple MdAGO4s, MdDRM2s and MdRDM1 were able to interact with each other *in vivo* and *in vitro*. Overexpression of *MdAGO4s* and *MdDRM2s* in apple callus led to increased transcript levels of the genes encoding their interacting proteins, suggesting that these three kinds of interacting proteins form an RdDM effector complex.

**Figure 7:**
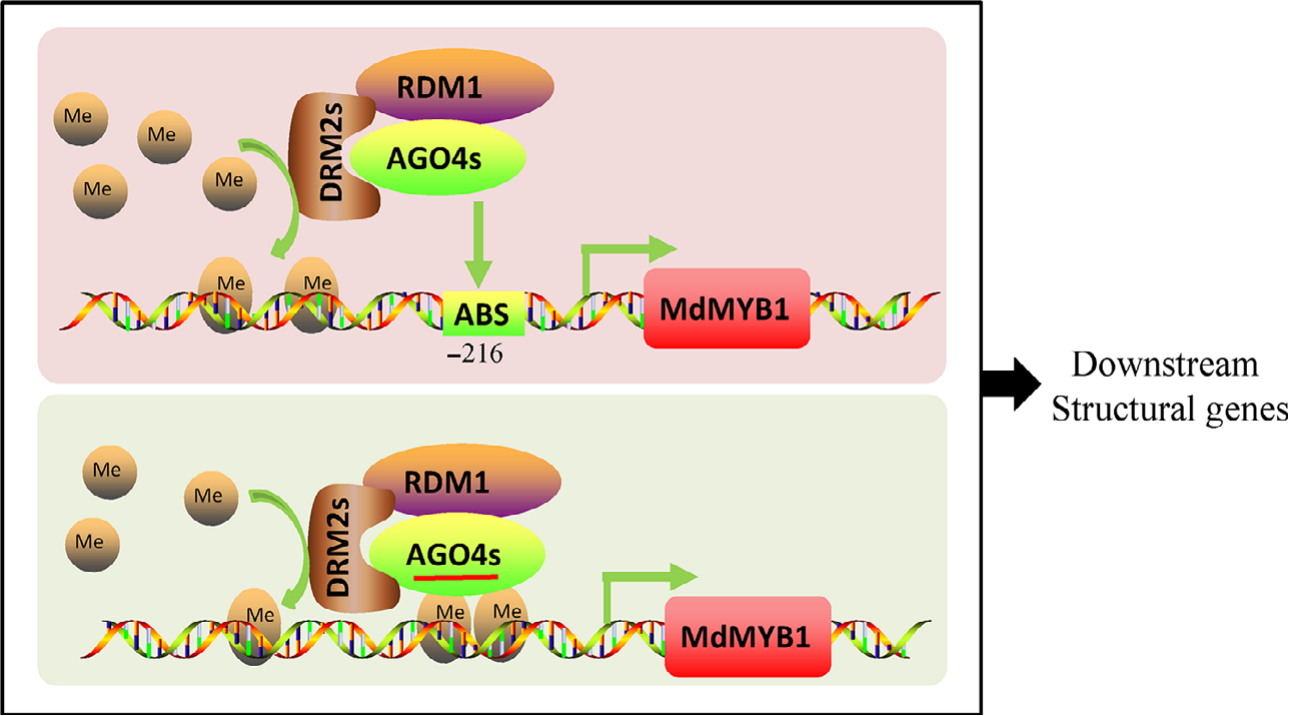
Potential model showing epigenetic modification of *MdMYB1* promoter through RdDM pathway. MdAGO4s, MdDRM2s and MdRDM1 interact with each other and form an effector complex. Upper box shows direct binding, where MdAGO4s bind to ATATCAGA sequence in *MdMYB1* promoter; lower box shows lncRNA‐mediated AGO4 binding. MdAGO4s recruit MdDRM2s, which catalyse CHH methylation of the *MdMYB1* promoter. MdMYB1 then regulates expression of downstream genes in anthocyanin pathway, thereby controlling anthocyanin accumulation to form different coloration patterns. AGO4, argonaute protein 4; DRM2s, domains rearranged methyltransferase2; RDM1, RNA‐directed DNA methylation1; MdMYB1, protein of MdMYB1; ABS, AGO4 binding sequence (ATATCAGA), brown balls, a ‐CH_3_ (methyl); solid arrow, direct regulation; *CHS*, chalcone synthase; *CHI*, chalcone isomerase; *F3H*, flavanone 3‐hydroxylase; *DFR*, dihydroflavonol 4‐reductase; *ANS*, anthocyanidin synthase; *UFGT*, flavonoid‐3‐O‐glucosyltransferase.

In *Arabidopsis*, both *ago4* (Zilberman *et al.*, 2003) and *rdm2* (Cao and Jacobsen, 2002) mutants showed lower CHH methylation at the *SUP* loci, indicating that AGO4 and DRM2 are essential for the maintenance of CHH methylation. In this study, in the *MdAGO4*‐ and *MdDRM2*‐overexpressing calli, the methylation level of the *MdMYB1* promoter was increased, indicating that the RdDM pathway is important for the formation of methylation. We further showed that heterologous expression of *MdAGO4s* and *MdDRM2s* in *Arabidopsis* mutants *ago4* and *drm2* was able to rescue the CHH methylation levels at several AGO4‐binding regions, which suggested that MdAGO4s and MdDRM2 are functionally conserved. Taken together, our analyses support that apple MdAGO4 and MdDRM2 form the RdDM effector complex to alter CHH methylation of AGO4‐binding sites (Figure 7).

### MdAGO4s bind to *MdMYB1* promoter

In plants, the maintenance of CHH methylation is controlled by DRM2, a domain‐rearranged methyltransferase in the RdDM pathway (Cao and Jacobsen, 2002). Other proteins in the RdDM pathway include AGO4, RDM1 and PolV (Wassenegger *et al.*, 1994; Wierzbicki *et al.*, 2009). In *Arabidopsis*, AGO4 preferentially targets the promoters of protein‐coding genes in the region between about −500 and −200 bp upstream of the transcription start sites (Zheng *et al.*, 2012). In this study, we found that MdAGO4 protein was able to bind to the promoter between −216 and −1 bp upstream of the transcription initiation site of *MdMYB1* in apple. We also found that MdAGO4s bound to the methylated region (MR3), where the DNA methylation differed among the three apple cultivars. More interestingly, we found that MdAGO4s only bound to the p4 region in an Y1H assay and to probe2 of the c3 in EMSA (Figure 3b and Figure S13). To further validate binding, two extra probes were synthesized for EMSAs. The MdAGO4s bound to *MdFLS* with the ATATCAGA sequence, but not to the *MdGST* probe. The *MdMYB1* and *MdFLS* probes both contained ATATCAGA sequence. Further EMSAs indicated that MdAGO4s specifically bind to this ATATCAGA sequence.

In *Arabidopsis,* the binding of AGO4 to specific gene promoters is mediated by lncRNAs produced by PolⅤ (Wierzbicki *et al.*, 2009). NRPE1 is the largest subunit of PolV, so we knocked down *MdNRPE1* to eliminate lncRNA in 35S::*AGO4‐1*‐GFP and 35S::*AGO4‐2*‐GFP transgenic apple calli, and then checked binding at MR3 and c3 regions. In the ChIP‐qPCR analyses, there was no significant difference in binding at the c3 region between the 35S::*AGO4*‐GFP calli and the co‐transgenic calli, but binding at the MR3 region was inhibited (Figures 5b and 6c). Those results provided the evidence that MdAGO4s can directly bind to DNA, and supported the results of Lahmy *et al. *(2016). Comparing these results with those of previous studies, direct binding of AGO4 may be a new binding pattern. In our view, both direct binding and small RNA‐guided binding of AGO4 occur in plant (Figure 7). Further research should focus on the effect of direct binding to gene loci and the relationships between the two binding patterns.

## Materials and methods

### Plant materials and growth conditions

‘Yanfu 3’, ‘Yanfu 8’ and ‘Nagafu 2’ were grown in Yantai, Shandong Province, China. For each variety, fruits were bagged on 15 May 2016 (at 30 days after full bloom; DAFB) and bags were removed at 164 DAFB. Bags were removed at the time that rapid anthocyanin accumulation occurred. Samples were taken at five time points: 0, 4, 8, 12 and 16 DABR. At each time point, four fruits per variety with three biological replicates were collected. All fruit peels were immediately frozen in liquid nitrogen and stored at −80 °C until use.

### Measurement of anthocyanin content

Anthocyanins were extracted using two buffer systems: KCl buffer, pH 1.0 (0.025 m), and NaAc buffer, pH 4.5 (0.4 m). Total anthocyanins were extracted from 0.5 g finely ground fruit peels in 5 mL 1% HCl in methanol (v/v) for 24 h at 4 °C in darkness. The first 1‐mL aliquot (three replicates) of the fruit peel extract was transferred to a 10‐mL centrifuge tube, and 4 mL KCl buffer was added. The second 1 mL aliquot (three replicates) of the fruit peel extract was placed in a 10‐mL centrifuge tube, and 4 mL NaAc buffer was added. Both solutions were mixed and extracted for 15 min at 4 °C in darkness. The absorbance of the solution was measured using a spectrophotometer (UV‐1600, Shimadzu, Kyoto, Japan) at 510 and 700 nm. The anthocyanin content formula was as follows: OD = (A_530_ − A_620_) − 0.1 × (OD_650_ − OD_620_).

### RNA isolation and RT‐PCR analysis

Total RNA was isolated using an RNAprep Pure Plant Kit (Tiangen, Beijing, China). First‐strand cDNA was synthesized using a TransScript II One‐Step gDNA Removal and cDNA Synthesis SuperMix Kit (TransGen, Beijing, China). The primers used for RT‐PCR were designed with Beacon Designer 7 and synthesized by the Sangon Biotech Co. (Shanghai, China) (Table S1). The RT‐PCRs were performed using cDNAs as templates with qPCR SuperMix (TransGen). Three biological and three technical replicates for each reaction were analysed on a CFX96 instrument (Bio‐Rad, Hercules, CA). The thermal cycling conditions were as follows: 94 °C for 30 s followed by 40 cycles of 94 °C for 5 s, 58 °C for 15 s, and 72 °C for 10 s. A melting curve was produced for each sample at the end of each run. Transcript abundance was calculated using the cycle threshold (Ct) $2^{-\Delta \Delta C_{t}}$ method (Livak and Schmittgen, 2001).

### Sequence analysis of *MdMYB1*

The full‐length coding region and genomic DNA (including promoter) sequence of *MdMYB1* were isolated from ‘Nagafu 2’, ‘Yanfu 3’ and ‘Yanfu 8’ using the primers listed in Table S1. The PCR analyses were conducted using Phusion DNA polymerase, following the manufacturer's instructions (Thermo Scientific, Waltham, MA). The PCR products were purified using an EasyPure Quick Gel Extraction Kit (TransGen). Then, the DNA fragments from three independent replicates were cloned using the pEASY‐Blunt Zero Cloning Kit (TransGen), and at least six clones were sequenced by the Sangon Biotech Co. The promoter sequence was analysed using the tools at the Plant *cis*‐acting regulatory DNA elements (PLACE) database (http://bioinformatics.psb.ugent.be/webtools/plantcare/html/).

### DNA extraction and methylation assay

Genomic DNA was extracted from fruit peel using a DNeasy Plant Mini Kit (Qiagen, Hilden, Germany). The McrBC‐PCR method was used to analyse the methylation levels of the *MYB1* promoter region. In this analysis, 1 μg gDNA from fruit skin samples of ‘Nagafu 2’, ‘Yanfu 3’ and ‘Yanfu 8’ was digested with the methylation‐specific endonuclease enzyme McrBC (NEB, Singapore) according to the manufacturer's instructions. Three biological replicates (each with three technological replicates) were analysed for each sample. For the negative control, water was used instead of guanosine‐5′‐triphosphate (GTP) in the reaction. Semi‐quantitative PCR analysis was performed using the treated *g*DNAs as the template. The *MdMYB1* promoter sequence was divided into four fragments; each fragment was amplified with specific primers (Table S1). The amplicons were visualized by agarose gel (1%) electrophoresis and were used to evaluate the methylation levels of the corresponding regions.

The bisulphite sequencing PCR (BSP) analysis was carried out as described by Telias *et al. *(2011) with three biological replicates. The EZ DNA Methylation‐Gold Kit (Zymo Research, Orange, CA) was used to treat 750 ng *g*DNA extracted from ‘Nagafu 2’, ‘Yanfu 3’ and ‘Yanfu 8’ fruit skin samples. Then, using degenerate primers (Table S1), *MdMYB1* promoter fragments were amplified using TaKaRa Ex Taq^®^ Hot Start Version (TaKaRa, Otsu, Japan) with the treated *g*DNA as the template. The promoter fragments were ligated into the pEASY‐Blunt Zero vector (TransGen) and then sequenced by Sangon Biotech. Each fragment with three independent PCR reactions generated 15 independent clones for sequencing. The results were analysed using the online software Kismeth (Gruntman *et al.*, 2008).

### Cloning of *MdAGO4s* (Argonaute 4) and *MdDRM2s* for phylogenetic analyses

The coding sequences (CDSs) of *MdAGO4‐1/2* (MD07G1052200, MD07G1052400) and *MdDRM2‐1/2* (MD17G1031900, MD09G1029900) were amplified from apple using Phusion DNA polymerase (Thermo Scientific) with the primers shown in Table S1. Phylogenetic and evolutionary analyses were conducted with MEGA5.1 with 1000 bootstrap replicates (Kumar *et al.*, 2004).

### Yeast one‐hybrid assays

The CDS of *MdAGO4‐1/2* was recombined into the pGADT7 vector (Clontech, Palo Alto, CA), and the *MdMYB1* promoter sequence was inserted into the pHIS2 vector (Clontech). The primers used to amplify the CDS and promoter fragments are listed in Table S1. To determine the suitable concentration of 3‐amino‐1,2,4‐triazole(3‐AT) to suppress background histidine leakiness of the pHIS2 vector, the yeast strain Y187 transformed with each recombinant pHIS2 vector was grown on ‐Trp/‐His (‐T/‐H) media containing 3‐AT at different concentrations. The interactions between the *MdAGO4‐1/2* and four promoter fragments were detected on ‐Trp/‐Leu/‐His (‐T/‐L/‐H) medium containing 3‐AT at the suitable concentration for each vector construct. The empty pGADT7 vector was used as the control.

### Co‐immunoprecipitation assays

*MdAGO4‐1/2* and *MdDRM2‐1/2* were recombined into the pHBT‐AvrRpm1‐HA vector containing the haemagglutinin (HA) tag sequence. The *DRM2‐1/2* and RNA‐directed DNA methylation (*RDM1*, MD16G1197500) genes were recombined into the pHBT‐AvrRpm1‐FLAG vector containing the FLAG tag sequence. Protoplasts derived from Orin apple callus were used for transient transfection. The vectors and protoplast isolation method were described previously (He *et al.*, 2006). Fifty microlitres (1800 ng/µL) of HA and FLAG tag plasmids were co‐transfected into 1 mL protoplasts, and the transfected protoplasts were incubated for 6 h at room temperature. After vigorous vortexing and a brief spin, the supernatant was transferred into a 10‐mL tube with 20 µL anti‐HA agarose beads (Sigma‐Aldrich, St Louis, MO, and Germany) and incubated for another 3 h. The beads were washed four times with IP buffer, and then, Western blotting was performed to detect the beads using anti‐HA and anti‐FLAG antibodies.

### Pull‐down assays

The CDSs of *MdAGO4‐1/2* and *MdDRM2‐1/2* were cloned into the pET‐32a (+) vector (EMD Biosciences, Novagen), which contains a HIS‐tag sequence, and the *MdDRM2‐1/2* and *MdRDM1* genes were cloned into the pGEX‐4T‐1 vector (GE Healthcare Life Sciences, Boston, MA), which contains a GST‐tag sequence. The recombinant plasmids were transformed into *Escherichia coli* BL21 (TransGen) to produce fusion proteins. The pull‐down assay was performed using a His‐tagged Protein Purification Kit (CW Biotech, Beijing, China). The proteins were mixed with isopycnic binding buffer, added to Ni‐agarose resin and then incubated at 4°C for more than 12 h. After elution, Western blotting was performed to detect the eluted products with anti‐His and anti‐GST antibodies (Abmart, Shanghai, China).

### Chromatin immunoprecipitation qPCR assays

Chromatin immunoprecipitation (ChIP) assays were performed using the EZ ChIP^TM^ Chromatin Immunoprecipitation Kit (Upstate, Waltham, MA) according to the manufacturer's instructions. An anti‐GFP antibody (Abmart) was used for ChIP. The resultant samples were analysed by qPCR using the primers listed in Supplemental Table S1. This experiment was repeated three times.

### Electrophoretic mobility shift assays

Electrophoretic mobility shift assays (EMSAs) were conducted using the LightShift Chemiluminescent EMSA Kit (Thermo Scientific). *MdAGO4‐1/2* were cloned into the expression vector pET32a. The AGO4‐1/2 recombinant protein was expressed in *E. coli* strain BL21 (DE3) and purified using a Ni‐agarose His‐Tagged Protein Purification Kit (CW Biotech). All promoter probes were synthesized and labelled by the Sangon Biotech Co. Double‐stranded probes were synthesized using annealing buffer for DNA oligos (Beyotime, Shanghai, China). The partial primers used for EMSAs are listed in Table S1.

### Mutation experiment of MdAGO4s

Mutations of MdAGO4s were performed as described previously (Cui *et al.*, 2013). The sequence of MdAGO4‐1‐m1, MdAGO4‐1‐m2, MdAGO4‐2‐m1 and MdAGO4‐2‐m2 (Figure S16a) was cloned used the primers listed in Table S1. Then, the mutated MdAGO4s were inserted to the pET32a vector to generate fusion protein.

### Transformation of apple callus

For gene transformation, *MdAGO4‐1/2* and *MdDRM2‐1/2* were each cloned into the pRI101‐AN vector (TaKaRa) containing a 35S promoter and a GFP tag sequence to construct the 35S::*AGO4‐1/2*‐GFP and 35S::*DRM2‐1/2*‐GFP recombinant vectors, respectively. For RNAi assays, the partial CDS of *MdNRPE1* was cloned into the pFGC1008 vector (http://www.chromdb.org) using the restriction sites *AscI*/*SwaI* and *BamHI*/*SpeI* for forward and reverse cloning, respectively. These vectors were then transformed into *Agrobacterium tumefaciens* LBA4404 competent cells. The transformed *Agrobacterium* cells were incubated with 2‐week‐old apple callus on MS medium without antibiotics for 48 h in the dark at 24 °C. Then, the apple callus was transferred to medium containing kanamycin and carbenicillin to select cells harbouring the genes.

### Northern blot analysis

Northern blotting was carried out using the protocol described by Streit *et al *(2008). Total RNAs were isolated from the apple callus using RNAprep Pure Plant Kit (Tiangen) and then separated by agarose gel electrophoresis. The separated RNA was transferred to Nylon membrane (GE Healthcare). ADNA fragment labelled with ^32^P was used for hybridization. Signals were captured by a phosphor screen, which was scanned using a Typhoon FLA 9500 scanner (GE Healthcare).

### Quantification of individual siRNAs

Small RNAs were extracted using a miRcute miRNA Isolation Kit (Tiangen). The abundance of 24‐nt siRNAs was quantitatively detected using Custom TaqMan^TM^ Small RNA Assays (Thermo Fisher Scientific) as described previously (Zhang *et al*, 2014).

### Heterologous expression of *MdAGO4s* and *MdDRM2s *in *Arabidopsis*

For *Arabidopsis* transformation, 35S::*AGO4‐1/2*‐GFP and 35S::*DRM2‐1/2*‐GFP recombinant plasmids were each introduced into *ago4* and *drm2* mutants via *Agrobacterium* strain GV3101. Seeds from T1 transgenic plants were grown and selected on MS medium containing kanamycin. The resistant transgenic seedlings were used for further analyses.

### DNA methylation analysis of *Arabidopsis*

Genomic DNA was extracted from 2‐week‐old seedlings using the DNeasy Plant Mini Kit (Qiagen). Then, 100 ng genomic DNA was digested with 10 U *AluI*, *DdeI* and *HaeIII* (NEB) for 20 min. After heat inactivation of the enzyme, digested DNA was amplified by PCR using Taq DNA Polymerase (Tiangen). Sequences lacking *AluI* (*IGN5*), *DdeI* (*AT2G36490*) and *HaeIII* (*AT2G27860*) restriction sites were used as loading controls.

### Statistical analyses

Statistical analyses were conducted using SPSS 19.0 (SPSS, Chicago, IL). Variance and significant difference tests were performed to identify differences among means by one‐way ANOVA with Tukey's HSD method.

## Conflict of interest

The authors declare no conflicts of interest.

## Author contributions

X.C. and S.J. designed the research. N.W., M.C., H.X. and Z.Z. provided advice on suggestions of the manuscript. S.J., Q.S., Y.W., H.F., W.F., M.S. and J.Z. performed the experiments. S.J. and X.C. analysed the data. R.Z. and S.J. performed the mutation experiment. S.W. provided the apple cultivation data. S.J. wrote the paper. X.S. provided the samples. Z.F. read and edited the manuscript.

## Supporting information


**Figure S1** Expression profiles of genes in anthocyanin and RdDM pathway.
**Figure S2** Phylogenetic analyses of AGO protein family in apple.
**Figure S3** Phylogenetic analyses of DRM2 protein family in apple.
**Figure S4** Interactions among MdAGO4s, MdDRM2s, and MdRDM1 detected in co‐immunoprecipitation (Co‐IP) assays.
**Figure S5** Interactions among MdAGO4s, MdDRM2s, and MdRDM1 detected in pull‐down assays.
**Figure S6** Characterization of ‘Orin’ apple calli overexpressing MdAGO4s and MdDRM2s.
**Figure S7** Relative transcript levels of genes involved in anthocyanin pathway in apple calli.
**Figure S8** Relative transcript levels of AGO4s, DRM2s and RDM1 in apple calli.
**Figure S9** Details of methylation region in MdMYB1 promoter in Orin calli.
**Figure S10** Characterization of red‐flesh apple calli overexpressing MdAGO4s and MdDRM2s.
**Figure S11** Details of methylation region in MdMYB10 promoter in red‐flesh apple calli.
**Figure S12** Northern blotting and RT‐PCR to confirm RNA interference knock‐down of NRPE1 and to quantitate of siRNA.
**Figure S13** Analysis of between MdAGO4‐1/2 and promoter of MdMYB1 by electrophoretic mobility shift assays.
**Figure S14** Analysis of interaction between MdAGO4‐1/2 and promoter of MdGST and MdFLS by electrophoretic mobility shift assays.
**Figure S15** Electrophoretic mobility shift assays of interaction between MdAGO4s and labeled DNA probes for ATATCAGA sequence within MdMYB1 promoter.
**Figure S16** Specific DNA‐binding domain of MdAGO4s to MdMYB1 promoter.
**Table S1** Primers used this study.
**Table S2** Restriction sites of AluI, DdeI, and HaeIII in Arabidopsis.
**Table S3** lncRNAs targeted MdMYB1 in apple.
**Table S4** 24nt siRNA sequences.
