# Supplementary material for: Methylation of MdMYB1 locus mediated by RdDM pathway regulates anthocyanin biosynthesis in apple
Source: Plant Biotechnol J. 2020 Jan 30;18(8):1736–48. doi: 10.1111/pbi.13337 (PMC7336386; doi:10.1111/pbi.13337)
Supplement: Supplementary file 1 — Figure S1 Expression profiles of genes in anthocyanin and RdDM pathway. Figure S2 Phylogenetic analyses of AGO protein family in apple. Figure S3 Phylogenetic analyses of DRM2 protein family in apple. Figure S4 Interactions among MdAGO4s, MdDRM2s, and MdRDM1 detected in co‐immunoprecipitation (Co‐IP) assays. Figure S5 Interactions among MdAGO4s, MdDRM2s, and MdRDM1 detected in pull‐down assays. Figure S6 Characterization of ‘Orin’ apple calli overexpressing MdAGO4s and MdDRM2s. Figure S7 Relative transcript levels of genes involved in anthocyanin pathway in apple calli. Figure S8 Relative transcript levels of AGO4s, DRM2s and RDM1 in apple calli. Figure S9 Details of methylation region in MdMYB1 promoter in Orin calli. Figure S10 Characterization of red‐flesh apple calli overexpressing MdAGO4s and MdDRM2s. Figure S11 Details of methylation region in MdMYB10 promoter in red‐flesh apple calli. Figure S12 Northern blotting and RT‐PCR to confirm RNA interference knock‐down of NRPE1 and to quantitate of siRNA. Figure S13 Analysis of between MdAGO4‐1/2 and promoter of MdMYB1 by electrophoretic mobility shift assays. Figure S14 Analysis of interaction between MdAGO4‐1/2 and promoter of MdGST and MdFLS by electrophoretic mobility shift assays. Figure S15 Electrophoretic mobility shift assays of interaction between MdAGO4s and labeled DNA probes for ATATCAGA sequence within MdMYB1 promoter. Figure S16 Specific DNA‐binding domain of MdAGO4s to MdMYB1 promoter. Table S1 Primers used this study. Table S2 Restriction sites of AluI, DdeI, and HaeIII in Arabidopsis. Table S3 lncRNAs targeted MdMYB1 in apple. Table S4 24nt siRNA sequences. [file PBI-18-1736-s001.docx]

**
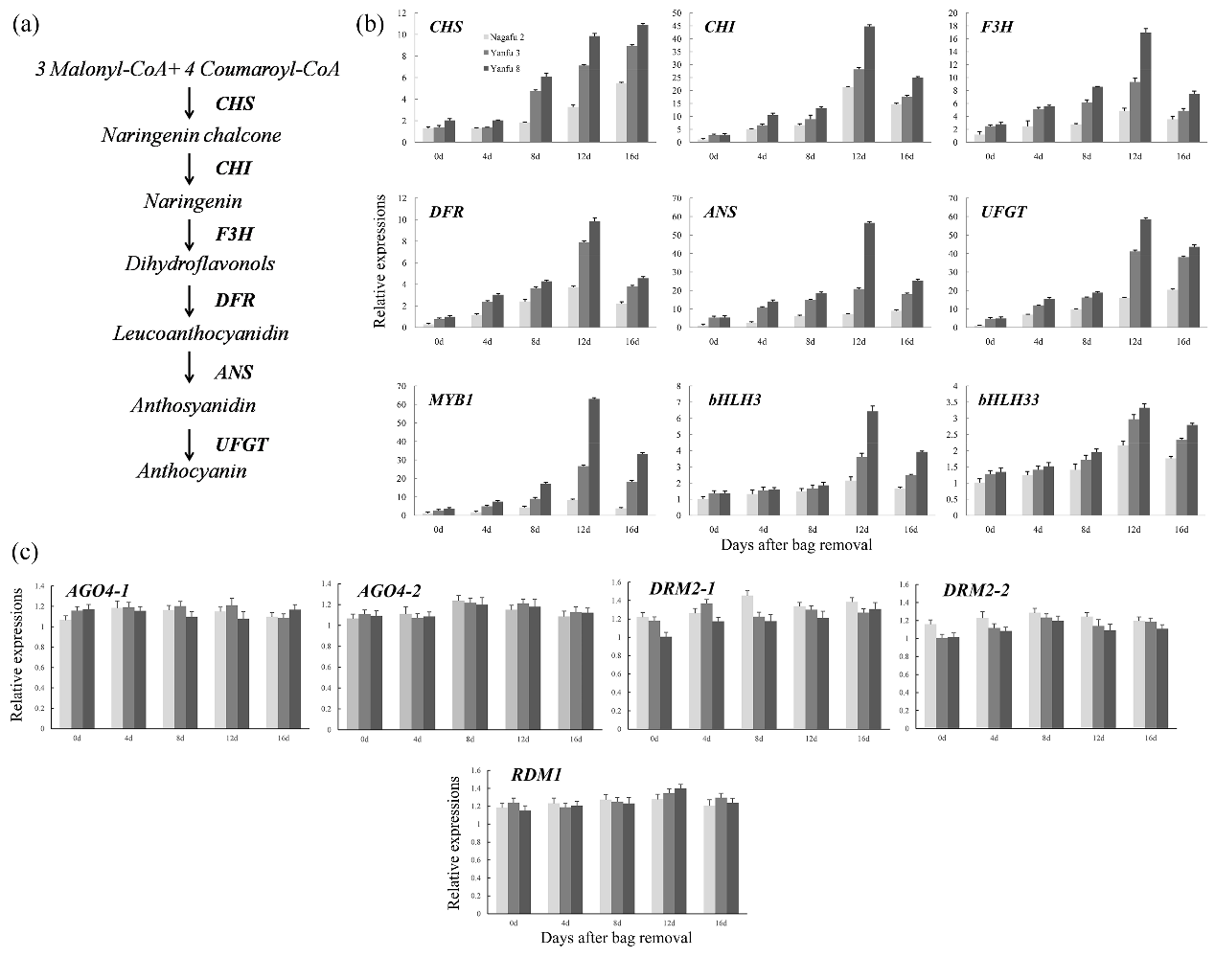
Figure S1 Expression profiles of** **genes of anthocyanin and RdDM pathway.**

(a) Anthocyanin biosynthetic pathway in apple. *CHS*, chalcone synthase; *CHI*, chalcone isomerase; *F3H*, flavanone 3-hydroxylase; *DFR*, dihydroflavonol 4-reductase; *ANS*, anthocyanidin synthase; *UFGT*, flavonoid-3-O-glucosyltransferase. (b) Relative transcript levels of anthocyanin biosynthetic and regulatory genes during fruit development after bag removal in ‘Nagafu 2’, ‘Yanfu 3’, and ‘Yanfu 8’. (c) Relative transcript levels of genes of RdDM pathway during fruit development after bag removal in ‘Nagafu 2’, ‘Yanfu 3’, and ‘Yanfu 8’.

**
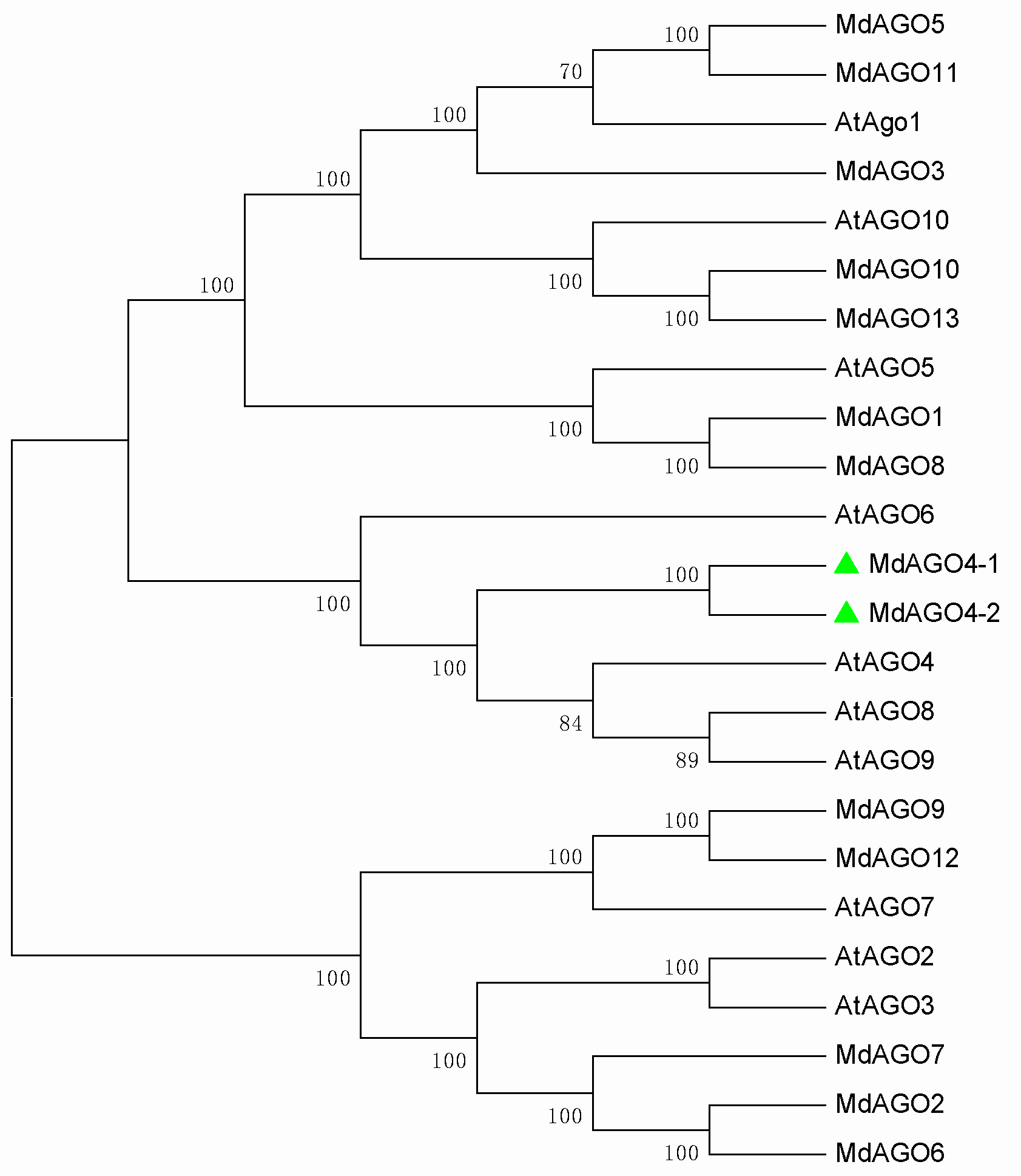
**

**Figure S2** **Phylogenetic analyses of AGO protein family in apple.**

Phylogenetic tree of homologous AGO family: AtAGO1, AT1G48410; AtAGO2, AT1G31280; AtAGO3, AT1G31290; AtAGO4, AT2G27040; AtAGO5, AT2G27880; AtAGO6, AT2G32940; AtAGO7, AT1G69440; AtAGO8, AT5G21030; AtAGO9, AT5G21150; AtAGO10, AT5G43810; MdAGO1, MD03G1130600; MdAGO2, MD05G1256100; MdAGO3, MD06G1237000; MdAGO4-1, MD07G1052200; MdAGO4-2, MD07G1052400; MdAGO5, MD06G1237100; MdAGO6, MD10G1236300; MdAGO7, MD10G1236400; MdAGO8, MD11G1152200; MdAGO9, MD13G1068200; MdAGO10, MD13G1201400; MdAGO11, MD14G1243900; MdAGO12, MD16G1067000; MdAGO13, MD16G1201200.


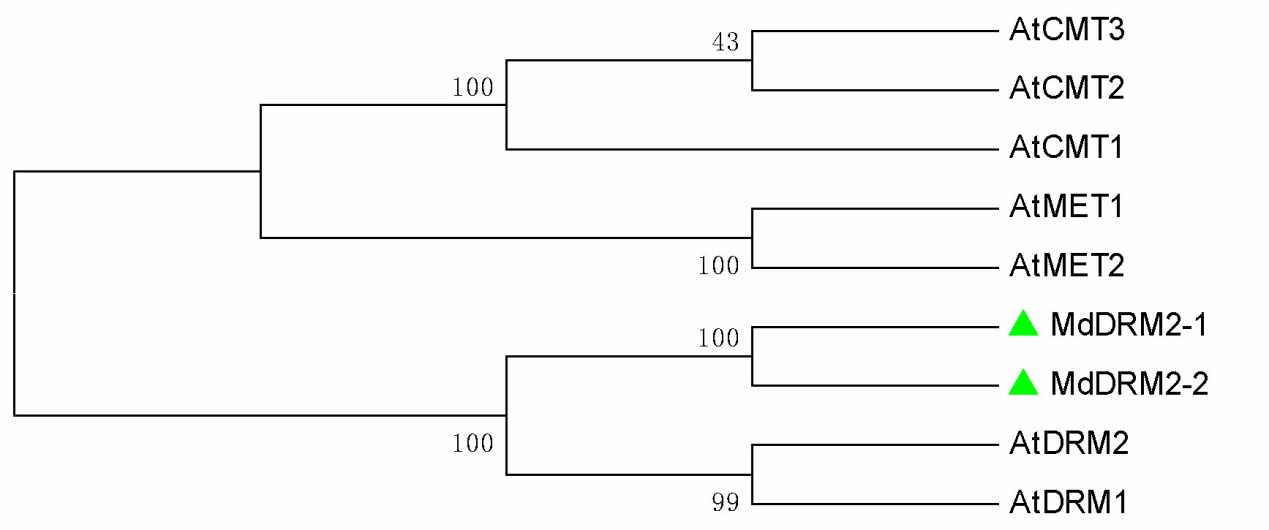


**Figure S3 Phylogenetic analyses of DRM2 protein family in apple.**

MdDRM2-1, MD17G1031900; MdDRM2-2, MD09G1029900; AtDRM1, AT5G15380; AtDRM2, AT5G14620; AtCMT1, AT1G80740; AtCMT2, AT4G19020; AtCMT3, AT1G69770; AtMET1, AT5G49160; AtMET2, AT4G14140.


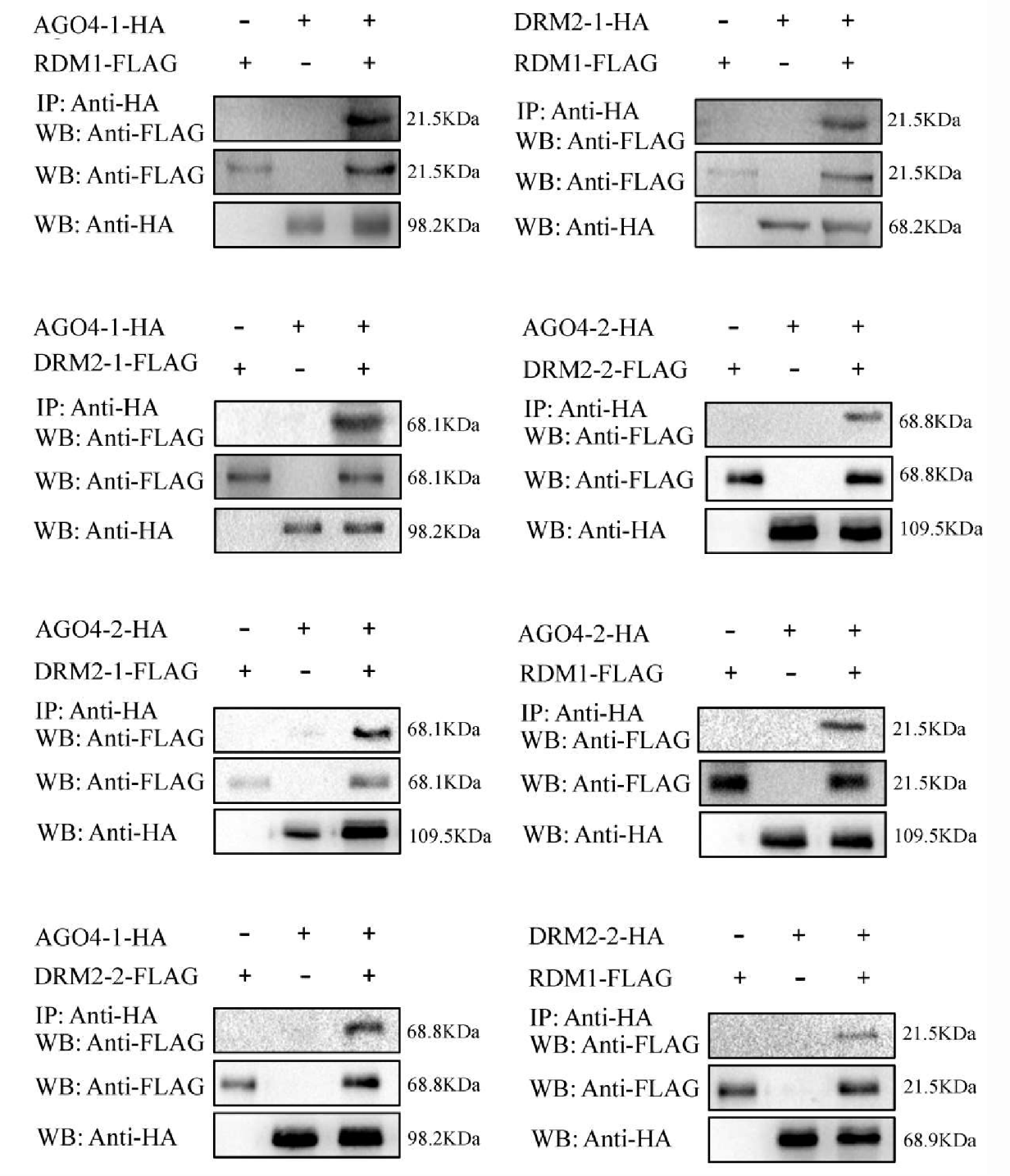


**Figure S4 Interactions among MdAGO4s, MdDRM2s, and MdRDM1** **detected in co-immunoprecipitation (Co-IP) assays.**

MdAGO4s-HA with MdDRM2s-FLAG, MdDRM2s-HA, and MdRDM1-FLAG, MdAGO4s-HA with MdRDM1-FLAG were transiently co-expressed in protoplasts of apple callus; HA and FLAG antibodies were used in immunoblot analysis. Band detected by HA antibody from total proteins of protoplasts indicates interaction of MdAGO4s and MdDRM2s, MdDRM2s and MdRDM1, MdAGO4s and MdRDM1. MdAGO4s interact with MdDRM2s; MdAGO4s interact with MdRDM1; MdDRM2s interact with MdRDM1.

**
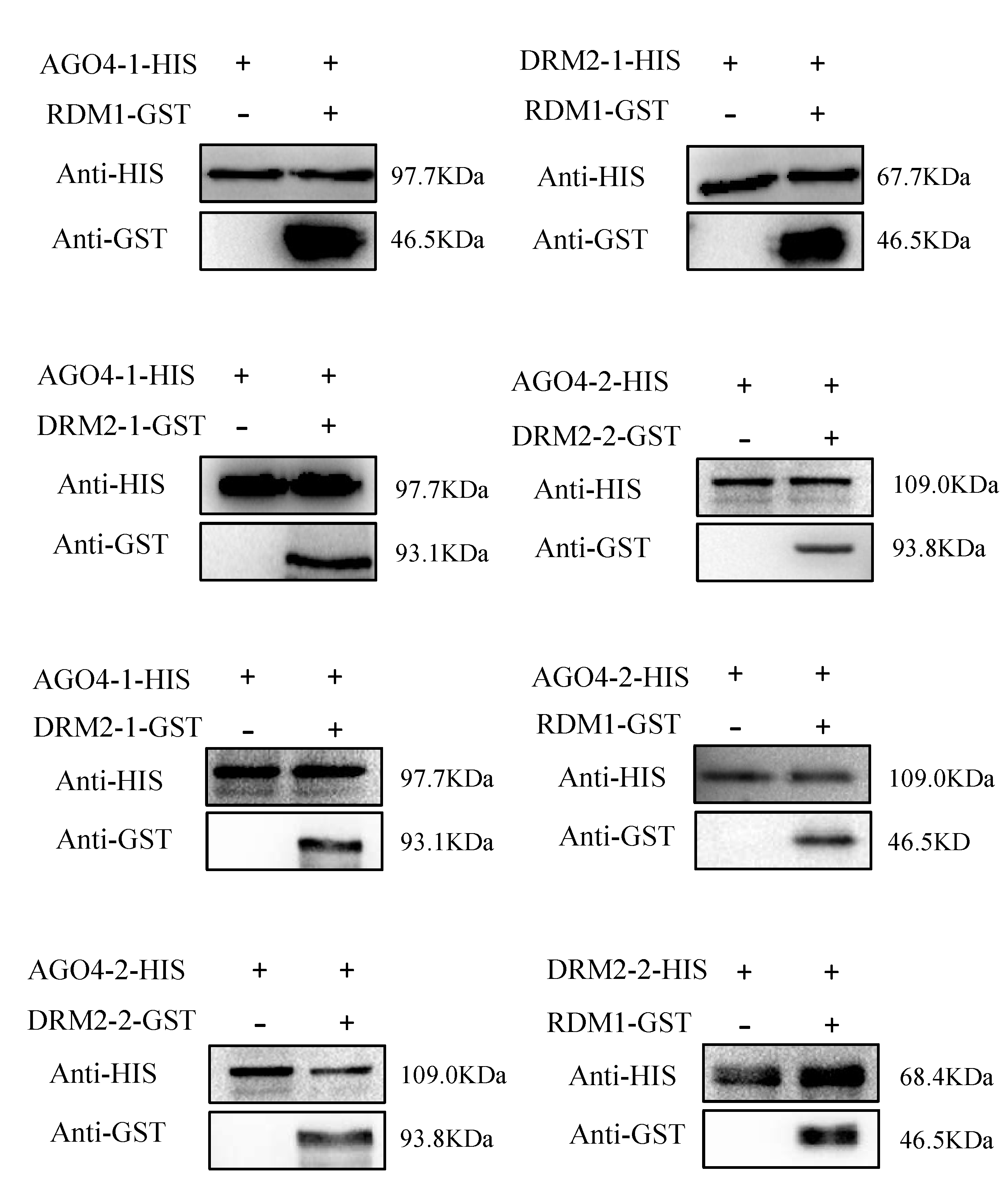
**

**Figure S5 Interactions among MdAGO4s, MdDRM2s, and MdRDM1 detected in pull-down assays.**

AGO4-1/2-HIS, DRM2-1/2-HIS, DRM2-1/2-GST and RDM1-GST fusion proteins were purified. Western blotting was using the GST antibody.


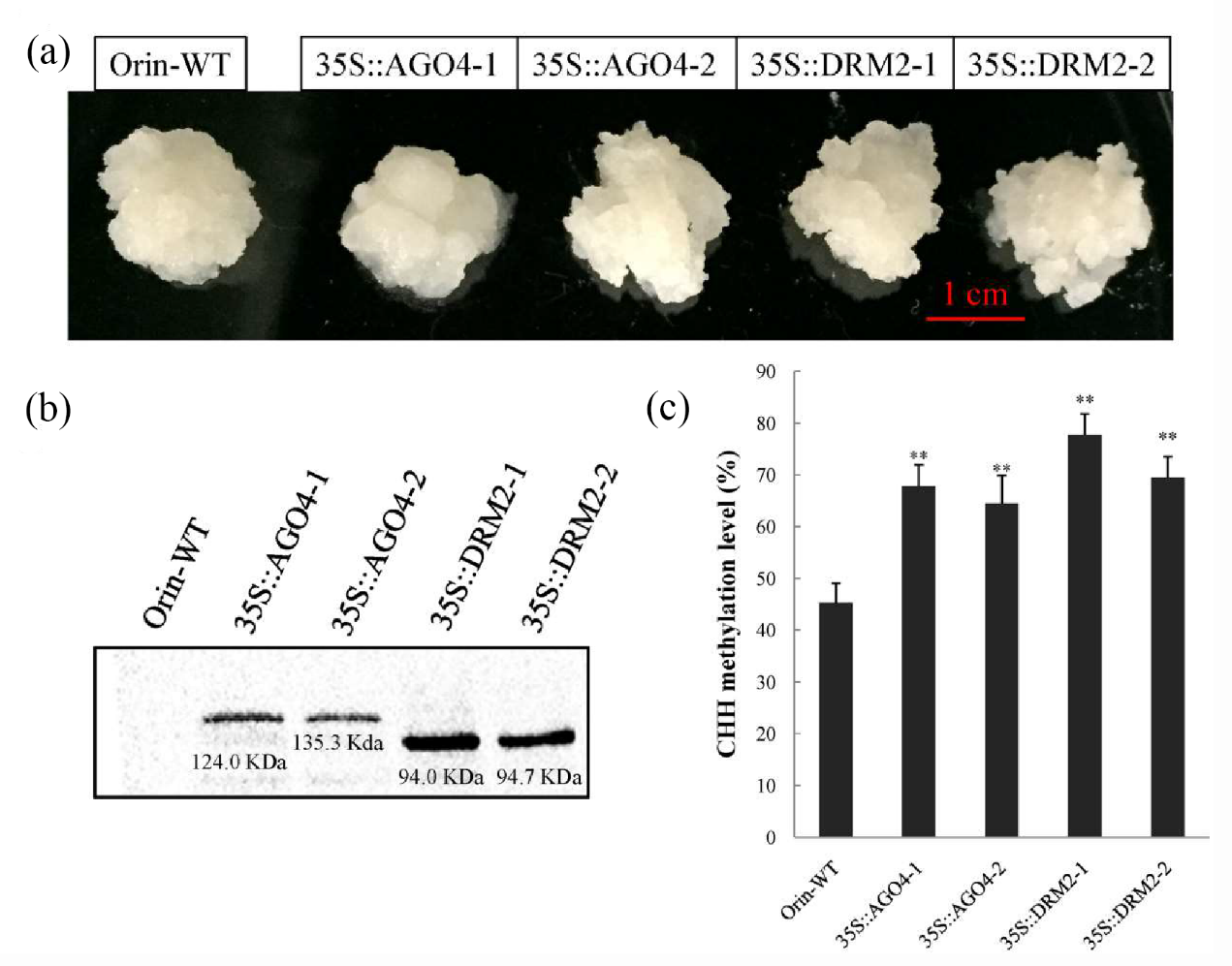


**Figure S6** **Characterization of ‘Orin’ apple calli overexpressing *MdAGO4s* and *MdDRM2s*.**

(a) Wild-type Orin callus (Orin-WT) and transgenic apple calli (35S::*AGO4-1/2* and 35S::*DRM2-1/2*) in natural growth states. (b) Expression of 35S::*AGO4-1/2* and 35S::*DRM2-1/2* in transgenic callus confirmed by western blotting with GFP antibody. Orin-WT was used as the negative control. (c) CHH methylation levels of the *MdMYB1* promoter (-1,657 to -1 bp) were determined using DNA extracted from transgenic calli by BSP. Error bars show s.d., ‘**’ indicates significance at *P* < 0.01.


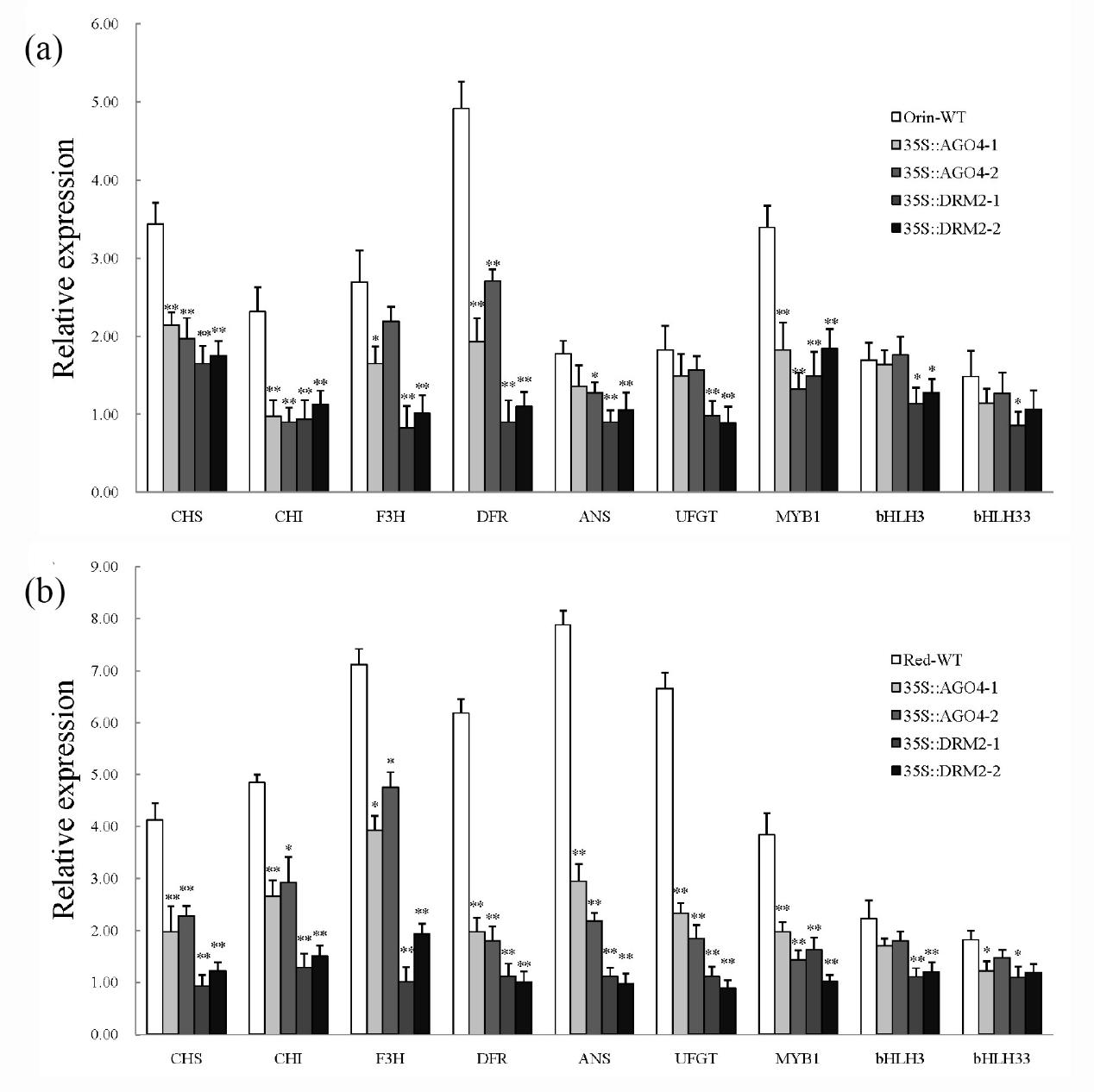


**Figure S7 Relative transcript levels of genes involved in the anthocyanin pathway in apple calli.**

Expression of genes was determined by qPCR analyses of 2-week-old calli in ‘Orin’ (a) and red-flesh apple callus(b). *CHS*, chalcone synthase; *CHI*, chalcone isomerase; *F3H*, flavanone 3-hydroxylase; *DFR*, dihydroflavonol 4-reductase; *ANS*, anthocyanidin synthase; *UFGT*, flavonoid-3-O-glucosyltransferase. Error bars show s.d., ‘*’ and ‘**’ indicate significance at *P* < 0.05 and *P* < 0.01, respectively.


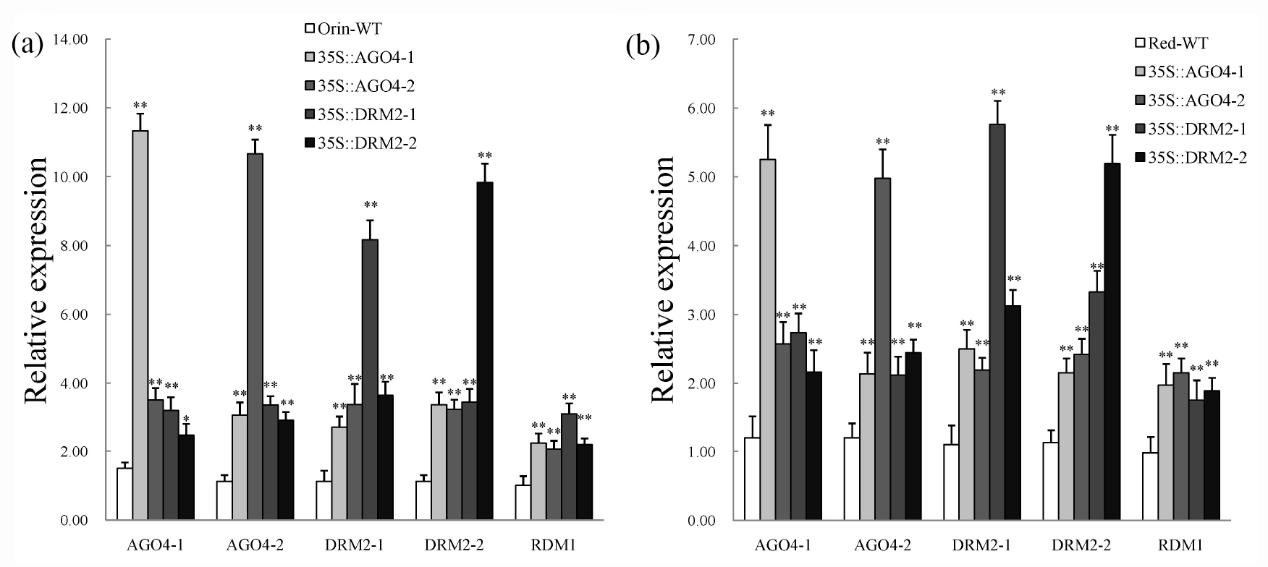


**Figure S8 Relative transcript levels of *AGO4s*, *DRM2s* and *RDM1* in apple calli.**

Expression of genes was determined by qPCR analyses of 2-week-old calli in ‘Orin’ (a) and red-flesh apple callus (b). AGO4, Argonaute protein4; DRM2, domains rearranged methyltransferase2; RMD1, RNA-directed DNA methylation1. Error bars show s.d., ‘**’ indicate significance at *P* < 0.01.


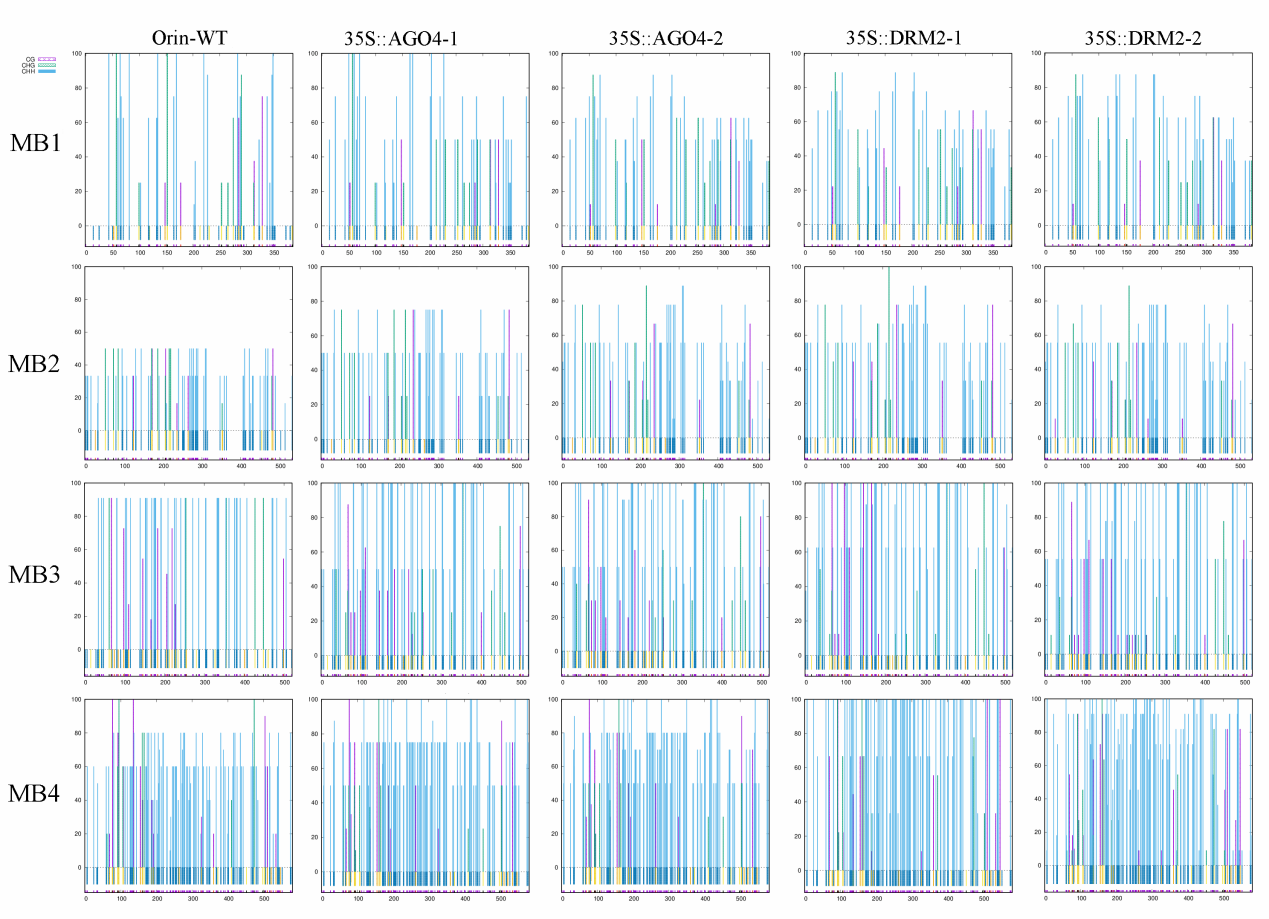


**Figure S9 Details of methylation region in *MdMYB1* promter in ‘Orin’ calli.**

The x axis indicates the position of each base and the y axis indicates the percent of methylated cytosines.

**
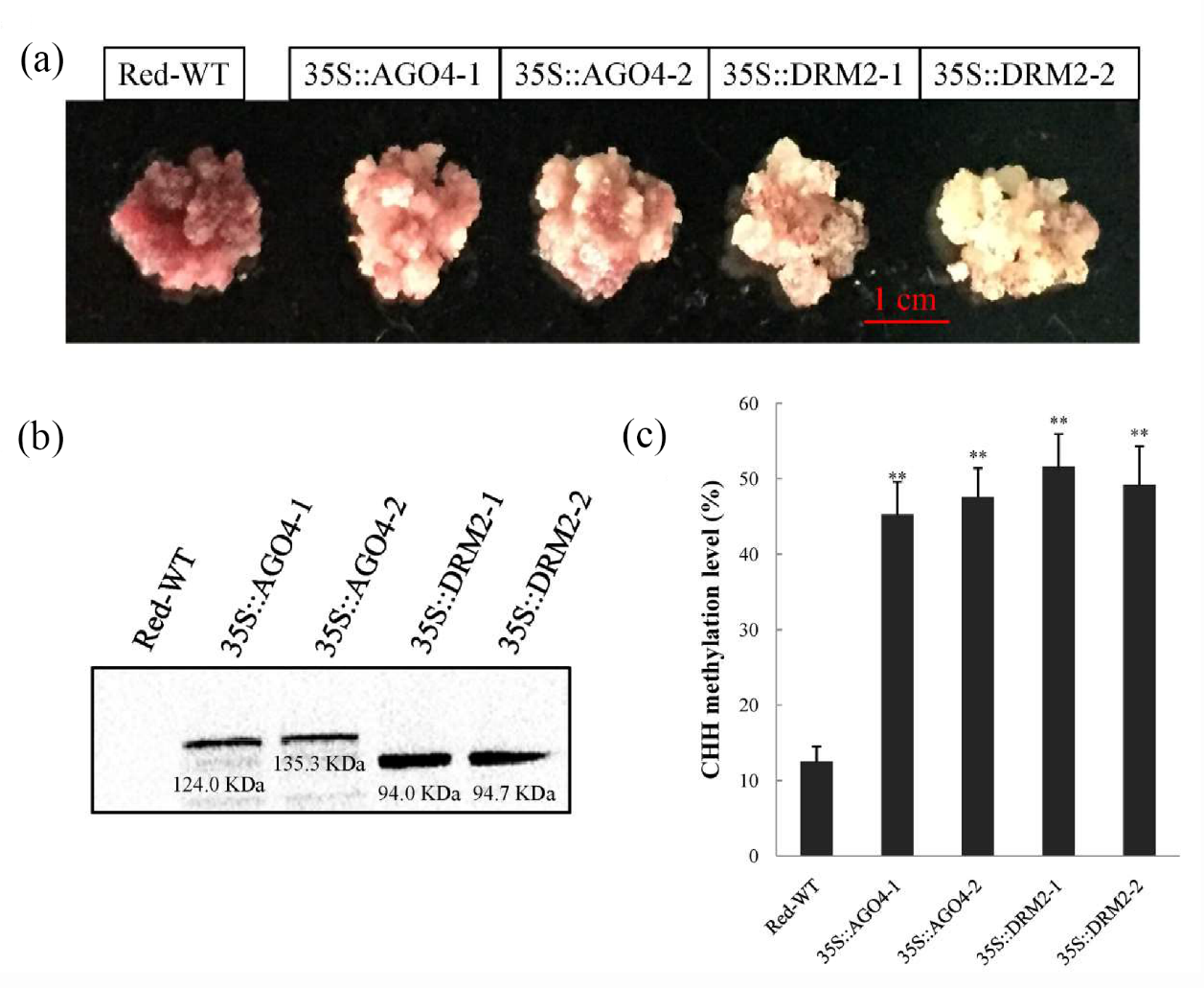
**

**Figure S10** **Characterization of red-flesh apple calli overexpressing *MdAGO4s* and *MdDRM2s*.**

(a) Wild-type red-flesh apple callus (Red-WT) and transgenic apple calli (35S::*AGO4-1/2* and 35S::*DRM2-1/2*). (b) Expression of 35S::*AGO4-1/2* and 35S::*DRM2-1/2* in transgenic calli confirmed by western blotting with GFP antibody. (c) Red-WT was used as the negative control. CHH methylation levels of the *MdMYB1* promoter (-1,758 to -1 bp) were determined using DNA extracted from transgenic calli by BSP. Error bars show s.d., ‘**’ indicate significance at *P* < 0.01.

**
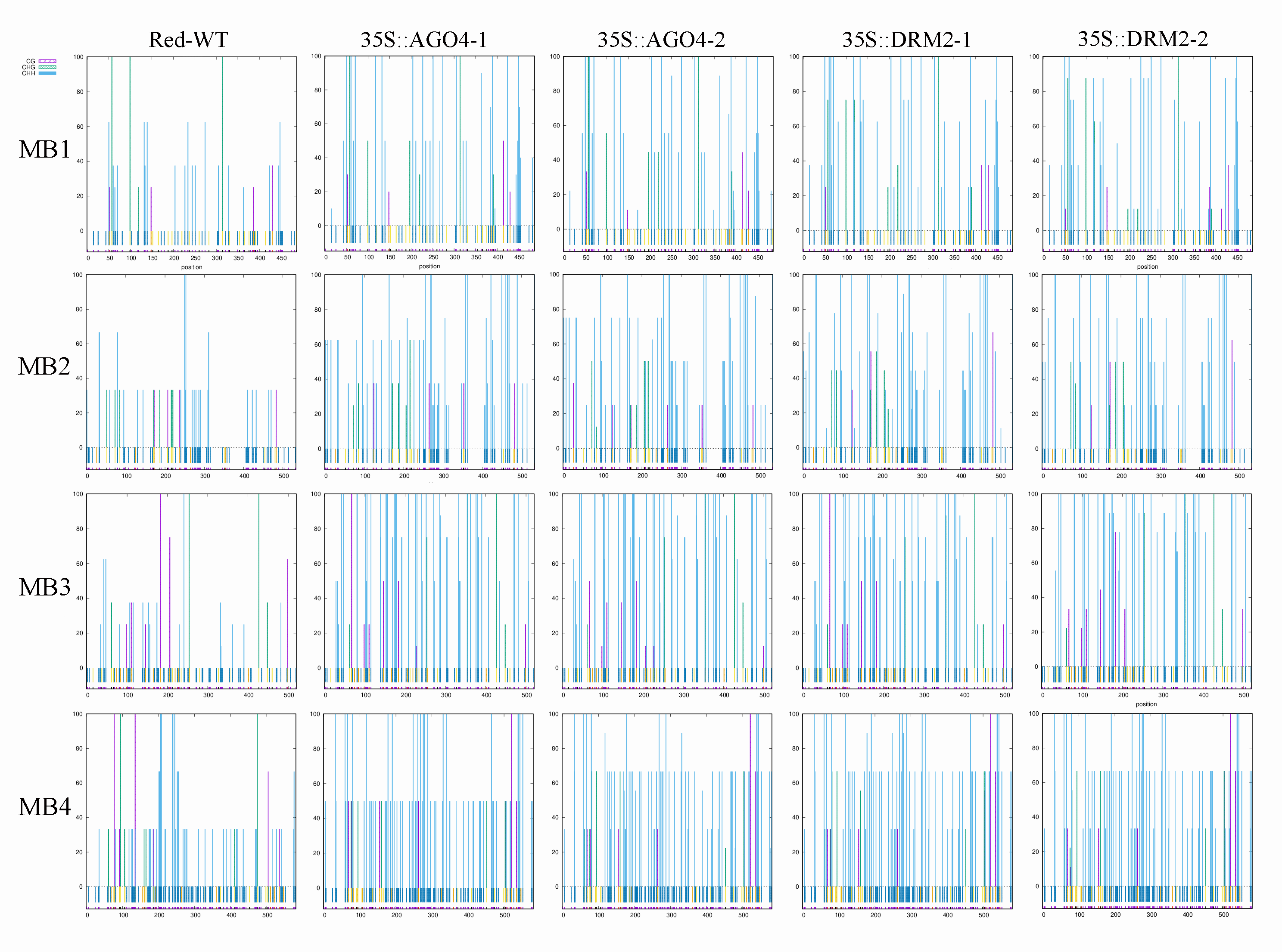
**

**Figure S11 Details of methylation region in *MdMYB10* promoter in Red-flesh apple calli.**

The x axis indicates the position of each base and the y axis indicates the percent of methylated cytosines.


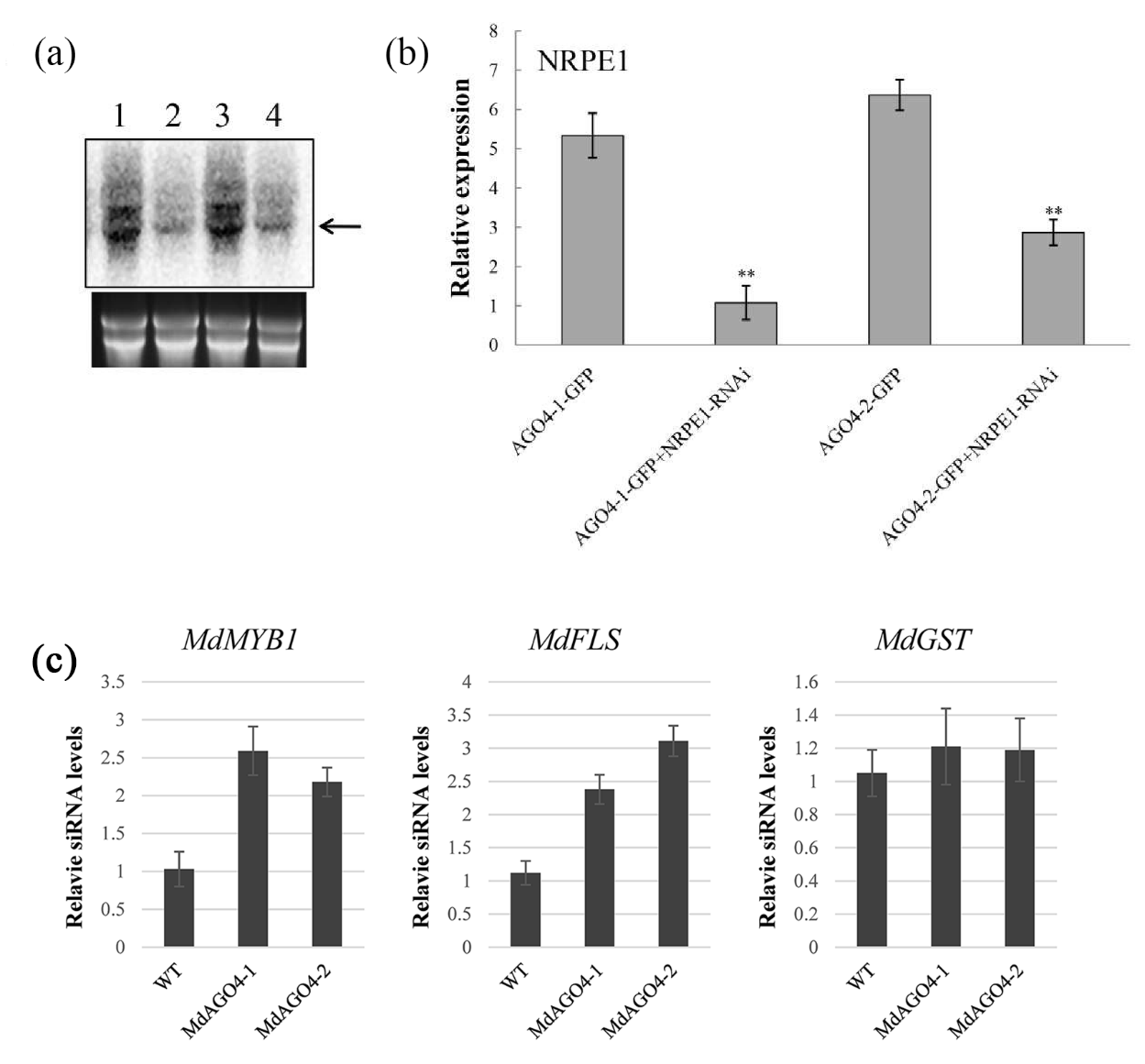


**Figure S12 Northern blotting and RT-PCR to confirm RNA interference knock-down of *NRPE1* and to quantitate of siRNA.**

(a) Northern blotting for testing the inhibition of *NRPE1* in the *NRPE1*-RNAi callus. Lane 1 indicates *AGO4-1*-GFP; lane2 indicate *AGO4-1*-GFP+*NRPE1*-RNAi; lane 3 indicates *AGO4-2*-GFP; lane 4 indicates *AGO4-2*-GFP+*NRPE1*-RNAi. (b) Expression profile of *NRPE1* in the *NRPE1*-RNAi calli. (c) Quantification of 24-nt siRNAs by TaqMan small RNA assays. Error bars show standard derivation of three replicates, ‘**’ indicates significance at *P* < 0.01.


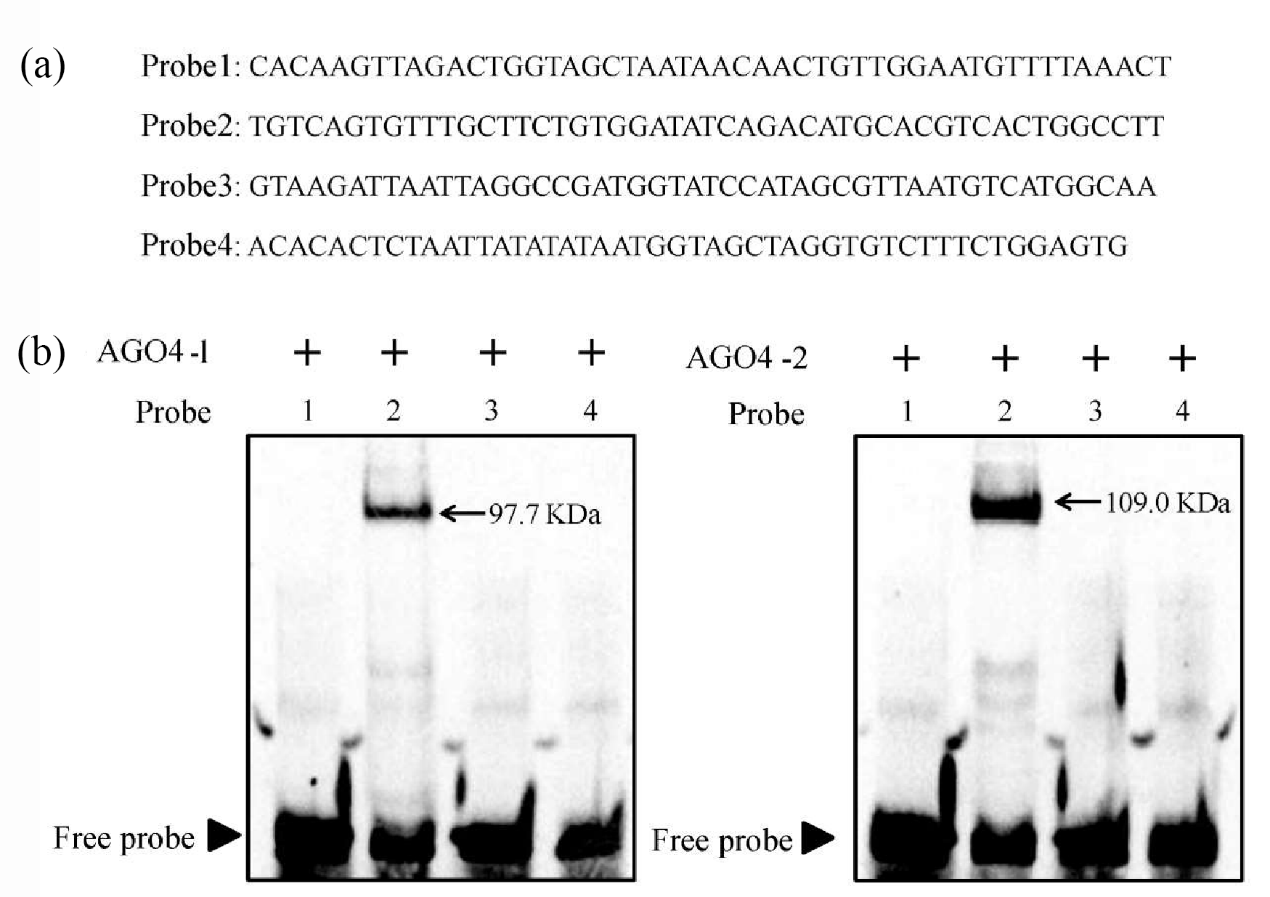
**Figure S13 Analysis of between MdAGO4-1/2 and promoter of *MdMYB1* by electrophoretic mobility shift assays.**

(a) Four sequences of *MdMYB1* promoter used in EMSAs. (b) EMSAs showing the binding of MdAGO4-l to different mutated probes of the Probe 2. *MdMYB1* without MdAGO4-1/2 binding site was used as a negative control. MdAGO4-1/2 were purified with His-tag. Probes used for EMSAs are all listed in Table S1.


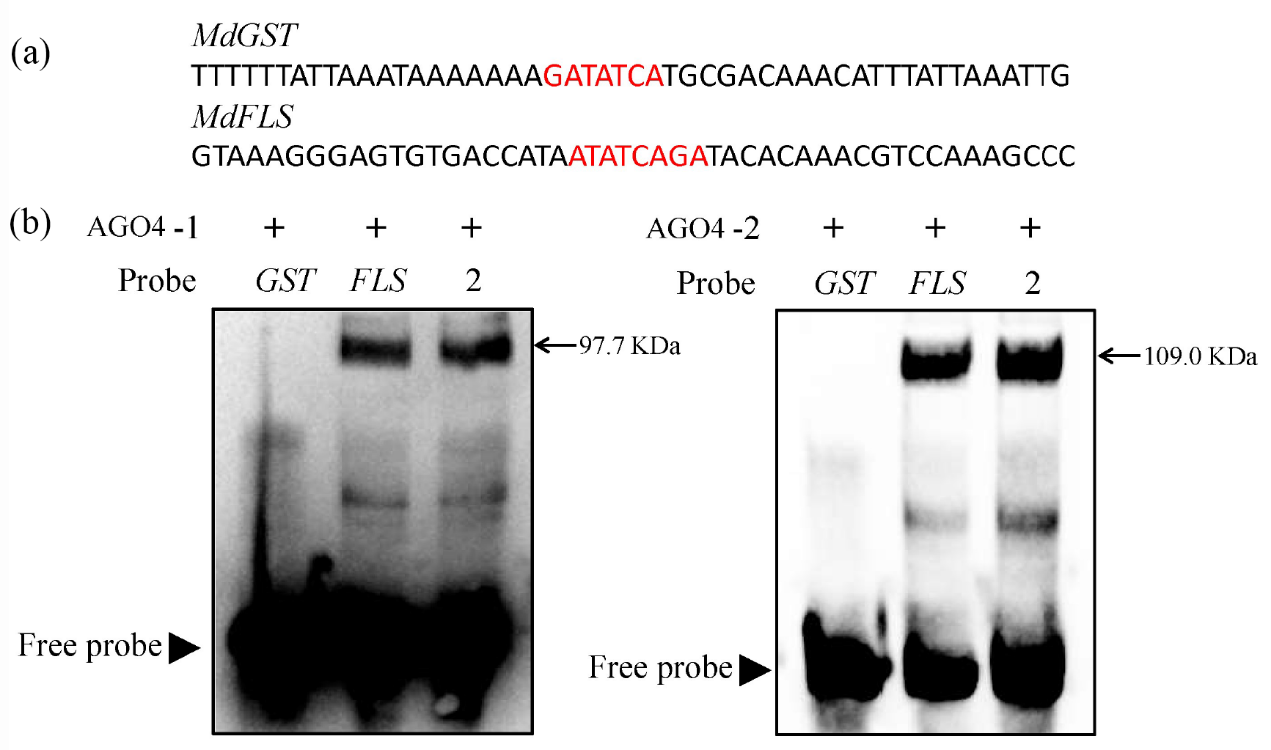


**Figure S14 Analysis of interaction between MdAGO4-1/2 and promoter of MdGST and MdFLS by electrophoretic mobility shift assays.**

(a) *MdGST* and *MdFLS* promoter probes used in EMSA. (b) Binding of MdAGO4-1/2 to the *MdFLS* promoter instead of *MdGST* promoter. Probe 2 was used as a positive control.


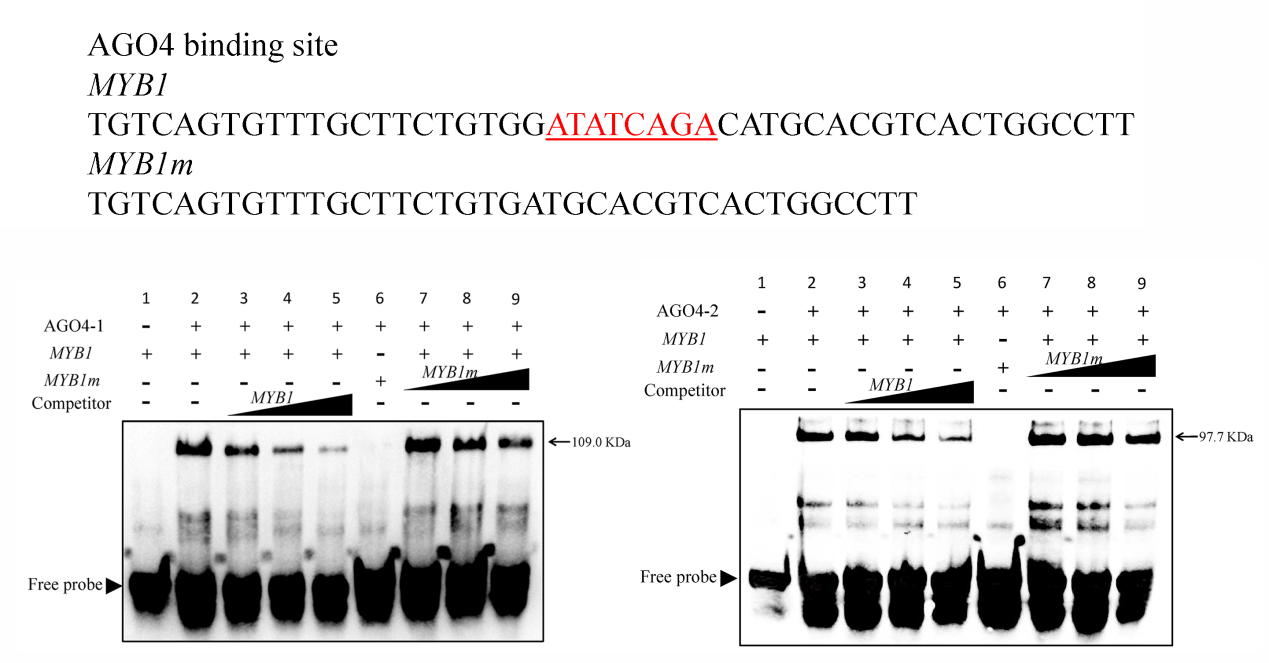


**Figure S15 Electrophoretic mobility shift assays of interaction between MdAGO4s and labeled DNA probes for ATATCAGA sequence within the *MdMYB1* promoter.**

The red letters indicate the AGO binding site and mutated AGO binding site (*MYB1m*) within the *MdMYB1* promoter. Lane 2 indicates the labeled normal DNA probes and the MdAGO4 protein without a competitor. Increasing amounts (5×, 15× and 25×) of the unlabelled normal DNA probes (*MdMYB1* in lanes 3, 4 and 5 of each blot) or the unlabelled mutants (*MdMYB1m* in lanes 7, 8 and 9 of each blot) were added as cold competitors. Lane 6 shows the labeled mutated DNA probe and the MdAGO4s protein indicating that MdAGO4s could not bind to the mutant probe.


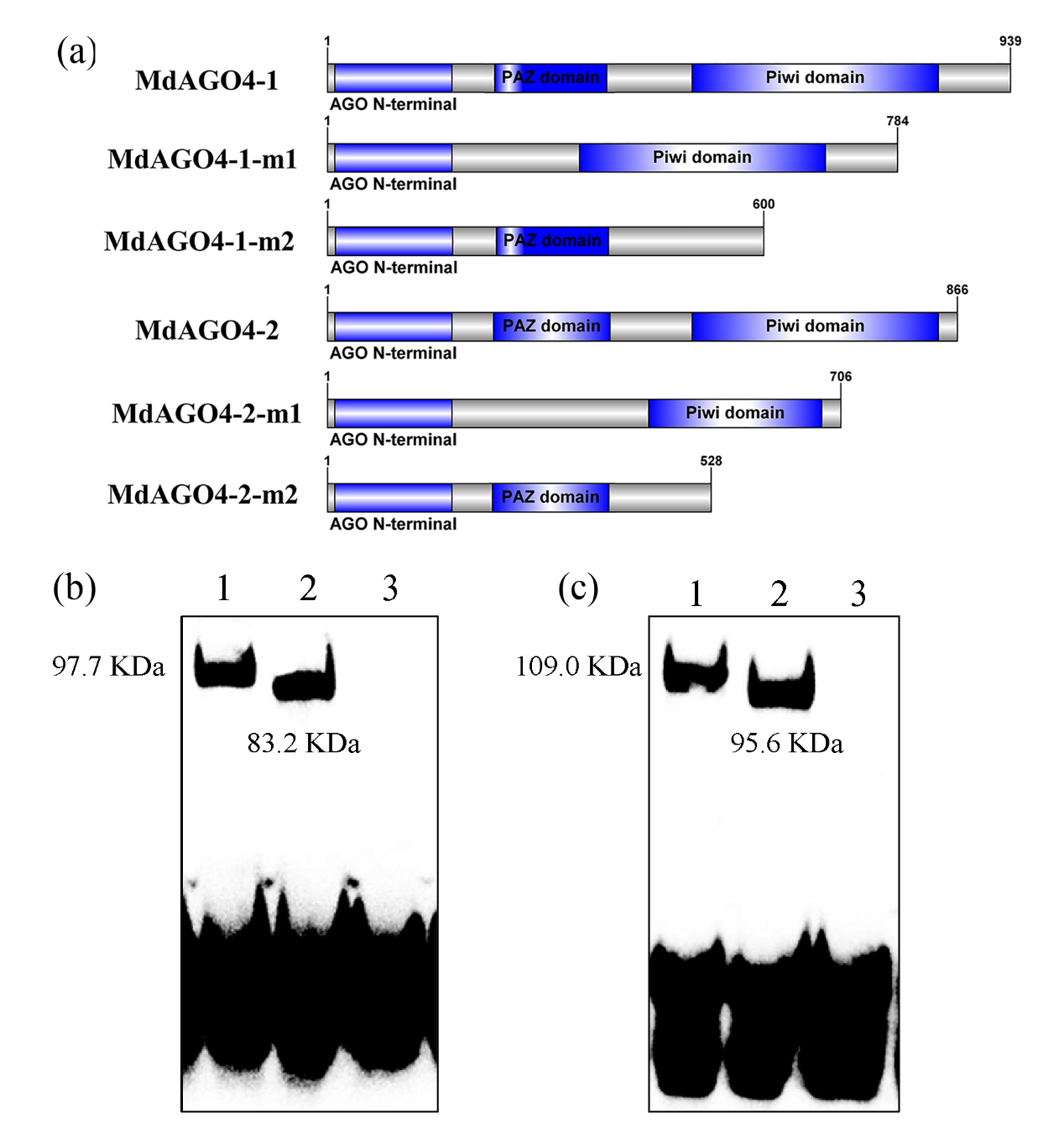


**Figure S16** **Specific DNA-binding domain of MdAGO4s to *MdMYB1* promoter.**

(a) The domain analysis of MdAGO4s and the deletion in MdAGO4s. (b) EMSA assays for testing MdAGO4-1 the binding to DNA without PAZ domain or Piwi domain. Line1, 2, and 3indicated MdAGO4-1, MdAGO4-1 without PAZ domain, and MdAGO4-1 without Piwi domain. (c) EMSAs for testing MdAGO4-1 the binding to DNA without PAZ domain or Piwi domain. Line1, 2, and 3indicated MdAGO4-2, MdAGO4-2 without PAZ domain, and MdAGO4-2 without Piwi domain. The probe 2 were used in this section.

| **Primers for quantative RT-PCR in apple**  **Table S1. Primers used in this study.** | | | | | |
| --- | --- | --- | --- | --- | --- |
| Gene name | Forward | | Reverse | | Accession number |
| CHS | GGAGACAACTGGAGAAGGACTGGAA | | CGACATTGATACTGGTGTCTTCA | | CN944824 |
| CHI | GGGATAACCTCGCGGCCAAA | | GCATCCATGCCGGAAGCTACAA | | CN946541 |
| F3H | TGGAAGCTTGTGAGGACTGGGGT | | CTCCTCCGATGGCAAATCAAAGA | | G104117 |
| DFR | GATAGGGTTTGAGTTCAAGTA | | TCTCCTCAGCAGCCTCAGTTTTCT | | AF117268 |
| ANS | CCAAGTGAAGCGGGTTGTGCT | | CAAAGCAGGCGGACAGGAGTAGC | | AF117269 |
| UFGT | CCACCGCCCTTCCAAACACTCT | | CACCCTTATGTTACGCGGCATGT | | AF117267 |
| MYB1 | TGCCTGGACTCGAGAGGAAGACA | | CCTGTTTCCCAAAAGCCTGTGAA | | AB744000 |
| bHLH3 | ACCACCTCAGCCAGAACCT | | CCTTCACCTTGGCTCTTAGTT | | KX822759 |
| bHLH33 | TCCGACAGAAGACTCCATGATG | | CGTGTTTAGCAAAAGAGTGAGCC | | DQ266451 |
| AGO4-1 | GAGAGTAACCAGCACAGA | | CTCAGGAAGAATACACAACAG | | MD07G1052200 |
| AGO4-2 | CTCGTCCGTGATCTGATC | | ACATCCTCTCAACTCTAACC | | MD07G1052400 |
| DRM2-1 | GGATGATGATGAGACAGTTC | | TAGTAGAAGTATGGAGGTCCTA | | MD17G1031900 |
| DRM2-2 | AGCGAGTCCAGAAGTATG | | GATATGCCACAGTGTCAAC | | MD09G1029900 |
| RDM1 | TCCTGTGCGTACTTATTCTA | | ATAACATCCACCTGCTCAT | | MD16G1197500 |
| ACTIN | TGACCGAATGAGCAAGGAAATTACT | | TACTCAGCTTTGGCAATCCACATC | | CN938023 |
| **Primers for sequence analysis of MdMYB1** | | | | | |
| Gene name | Forward | | Reverse | | Accession number |
| MYB1 promoter | GAATCCTAATAAAGATTTATAGGCAAAT | | CACTCCAGAAAGACACCTAGCTAC | | DQ886414 |
| MYB1 cds | ATGGAGGGATATAACGAAAACC | | TTCTTCTTTTGAATGATTCCAAAG | | DQ886414 |
| **Primers for McrBC** | | | | | |
| Fragment | | Forward | | Reverse | |
| MR1 | | GGTATCTTATGGTGGTCAAAGATG | | CTTATCTGCTAGCAGCTAAGCTTA | |
| MR2 | | CTGAGATTGACTCTTGTGAAAGCT | | GTGAATGCAGAATCGTGTAACTAGT | |
| MR3 | | TTAACGGAATCCAACGAAGACAAG | | GCTACACCTAACACATTGCTCAAT | |
| MR4 | | TGAGATAGGTCCGGTTCTATTTCT | | ATCCCTTTCCCTTATTTGTTCCGT | |
| **Primers for Bisulfite sequencing** | | | | | |
| Fragment | | Forward | | Reverse | |
| MB1 | | ATGATAAAGGTATTTATGGTGGTT | | CACTCCAGAAAGACACCTARCTAC | |
| MB2 | | TTTGYTTTAGGGGATAAATGATTT | | CTAATTACAACACACATCTTTAACCAC | |
| MB3 | | TTATGTATAAGAATTTGGGGTTTTGGA | | CTACACCTAACACATTACTCAATATAA | |
| MB4 | | TAATTTTAATAAAGATTTATAGGCAAATTAT | | TTAAATAATTTATATCCCTTTCCCTTA | |
| **Primers for gene cloning and transgene** | | | | | |
|  | | Forward | | Reverse | |
| AGO4-1 | | ATGGCAAGGAGTGGGAATG | | TTAACAGAAGAACATGGAGTTGATC | |
| AGO4-2 | | ATGGATTCGTTTGAGCCAG | | TCAACAGAAAAACATGGAACTGGAC | |
| DRM2-1 | | ATGGATGGTGAATACAGTAATGATT | | TCAATTGTTTCTTCCCATCATGCT | |
| DRM2-2 | | ATGGATGGTGAATACAGTAATGATTTT | | TCAATTGTTTCTTCCCATCATG | |
| 35S::AGO4-1 | | GTCGACATGGCAAGGAGTGGGAATG | | GGTACCACAGAAGAACATGGAGTTGATCAC | |
| 35S::AGO4-2 | | GTCGACATGGATTCGTTTGAGCCAG | | GGTACCTCAACAGAAAAACATGGAACTGG | |
| 35S::DRM2-1 | | CCCGGGATGGATGGTGAATACAGTAATGATT | | GAATTCATTGTTTCTTCCCATCATGCT | |
| 35S::DRM2-2 | | CCCGGGATGGATGGTGAATACAGTAATGATTTT | | GGTACCATTGTTTCTTCCCATCATGCTC | |
| NRPE1-F-RNAi | | GGCGCGCCTCAATGCTCAGAAAAGTTCTAAAG | | ATTTAAATAAGTGAGTAAGTTGATGATCCATC | |
| NRPE1-R-RNAi | | ACTAGTTCAATGCTCAGAAAAGTTCTAAAG | | GGATCCAAGTGAGTAAGTTGATGATCCATC | |
| **Primers for Y1H** | | | | | |
|  | | Forward | | Reverse | |
| AGO4-1-pGADT7 | | CATATGATGGCAAGGAGTGGGAATG | | GGTACCTTAACAGAAGAACATGGAGTTGATC | |
| AGO4-2-pGADT7 | | CATATGATGGATTCGTTTGAGCCAG | | GAGCTCTCAACAGAAAAACATGGAACTGGAC | |
| MYB1-pHIS2 | | GAATTCGAAAAAATTTAATAAAAAGGACCCT | | GAGCTCCACTCCAAAAAGACACCTAGCTAC | |
| p1-pHIS2 | | GAATTCGAAAAAATTTAATAAAAAGGACCCT | | GAGCTCGTAACTTAGGGTTACAATGCTAGACAATTG | |
| p2-pHIS2 | | GAATTCAGACACAAACATAAACTTGAGCAACTTCTATG | | GAGCTCTTCTCTCCTAAGATTTCAGGCTTACATTATTTTCATA | |
| p3-pHIS2 | | GAATTCTTCTTGCTCTAGGGGACAAATGATTTTCG | | GAGCTCTCATTCCCCTCCTATTTGAATAGTCACG | |
| p4-pHIS2 | | GAATTCAGAAGAGGGAAAAAAAGGAGAGAATCCTACT | | GAGCTCTCCACAGAAGCAAACACTGACAAGTTTAAAAC | |
| **Primers for Co-IP assay** | | | | | |
| Name | | Forward | | Reverse | |
| AGO4-1-HA | | GGATCCATGGCAAGGAGTGGGAATG | | CCCGGGACAGAAGAACATGGAGTTGATCAC | |
| AGO4-2-HA | | GGATCCATGGATTCGTTTGAGCCAG | | CCCGGGACAGAAAAACATGGAACTGGAC | |
| DRM2-1HA/FLAG | | GGATCCATGGATGGTGAATACAGTAATGATT | | CCCGGGATTGTTTCTTCCCATCATGCT | |
| DRM2-2HA/FLAG | | GGATCCATGGATGGTGAATACAGTAATGATTTT | | CCCGGGATTGTTTCTTCCCATCATGCTC | |
| RDM1-FLAG | | GGATCCATGGATCCCACACGTAAAAG | | AGGCCTCGATTTGCTTCGTAGCTGG | |
| **Primers for ChIP-qPCR assay** | | | | | |
| Name | | Forward | | Reverse | |
| c1 | | TGTACAGCAATATAGTGGGG | | TCATTCCCCTCCTATTTGA | |
| c2 | | AGGAGAGAATCCTACTCCATAA | | ATACACACACACACACACACTC | |
| c3 | | CGAGTGTGTGTGTGTGTGT | | CACTCCAGAAAGACACCTAGC | |
| MR3-1 | | CCGTAGGAGTTTTGTACAGCA | | TTCTCTCCTTTTTTTCCCTCTT | |
| MR3-2 | | TCCTACTCCATAAATTACAAGCAAA | | GTCCGAAACCTTGTCTTCG | |
| MR2 | | CACTCAACGGAACAAATAAGG | | CATGTGGTTAATCTACATTTAGGGT | |
| **Probes for EMSA assay** | | | | | |
| Name | | Forward | | Reverse | |
| FLS-prbe | | GTAAAGGGAGTGTGACCATAATATCAGATACACAA  ACGTCCAAAGCCC | | GGGCTTTGGACGTTTGTGTATCTGATATTATGGTC  ACACTCCCTTTAC | |
| GST-probe | | TTTTTTATTAAATAAAAAAAGATATCATGCGACAAA  CATTTATTAAATTG | | CAATTTAATAAATGTTTGTCGCATGATATCTTTTTT  TATTTAATAAAAAA | |
| **Primers for DNA methylation analysis of *Arabidopsis*** | | | | | |
| Locus | | Forward | | Reverse | |
| IGN5 | | AAGCCCAAACCATACACTAATAATCTAAT | | CCGAATAACAGCAAGTCCTTTTAATA | |
| AT4G31770 | | ATGCCAACGTTGACTCACGA | | TCATGAGTTTGGGAATGGTTTT | |
| AT3G48131 | | GGCAACAAGAAGTAGAGCAAATCG | | AAAAAGGAATGTGGAGAGATGAAA | |
| AT5G52070 | | GACTTAACGACGCGTATAATGTGG | | TGTGTGAATTAGCGAAACTTGTCA | |
| AT5G27860 | | TGGAAAAGAATTGAGAGAATGATCTTG | | TTCAACCTTTGTTGTTTATTTGTCCA | |
| AT4G08310 | | GGGTCGGGTTCGGTTAAAA | | CAAACCCGAACCCAAAACTAAA | |
| AT1G12730 | | TTCAAAGATGGAGTTTCACGTGTC | | CGACTTCCGTAACACCCATT | |
| AT2G01735 | | CAAATCTGAAGTCGAACCCAAAA | | GTCGGATTCGGGTAAAATTCG | |
| AT2G36490 | | GGAAACATGTCCAGCGCTTT | | TGGAAGAGAAGCAGTTTCAGCA | |
| AT3G30380 | | GAGGGAAAGATGATCCGTCAA | | TGCACAAAATGACTATGAATTGTAAA | |
| AT1G49490 | | TGAGGCTAAAATGATGATAAAATCCA | | AACCATGTCTCTGCATATTCAATC | |
| AT1G35160 | | TTGCTCAATCAACAGAAATTACAAAA | | TGAAAAAGGTGGAGAAAGAAAGAGA | |
| Primers for MdAGO4s deletion | | | | | |
| MdAGO4-1-m1 | | GATGCTGACCTGATGCTTC | | TGTAGTGGATACGTCCATATTAAGAG | |
| MdAGO4-1-m2 | | TCCACCACCATGGCTTG | | GAAGTTTTGACTTTATGTATTCAAACAT | |
| MdAGO4-2-m1 | | GCTGACCGGATGCTTCA | | TGTAGTGGATACATCCATGTTCAG | |
| MdAGO4-2-m2 | | GGCACCGCCACACC | | AAGAATACACAGCAGAAGCTTTG | |

Table S2. Restriction sites of *AluI*, *DdeI*, and *HaeIII* in *Arabidopsis.*

> AT4G31770 (348 bp) *AluI* site

TCATGAGTTTGGGAATGGTTTTAACCAACTTATAATGAATAGAGATACGGTATATGTTTTTTATATACTGGATTGATATTTTAGCTCAATTCTCTCAACCAAAAGATTTTCCGCCTTAGGCTTATAGTACTTCGGACCCGATCCAAGCCACATGGTATAATACTATAGATATAGATAGTCTACATGGGTTCTTTGGTAAAATGATTCATTAAACAGCGATTTTTCTTTTTTTCTTAAACAACCGATTCTTGTATCTGAAAAAGAGTTATGAAAAAACGAAGTATAATCCGACTCAGACGATTTATCGAATTCATTAAGATTTCAAAACTCGTGAGTCAACGTTGGCAT

> AT3G48131 (298 bp) *AluI* site

AAAAAGGAATGTGGAGAGATGAAACTTTTTTTTAAATAATAATTTCTTAATTTTCAGGGGCAAATTGGTCAAAAGTTTAATGAAAAAGTTGGAAAAGTGCCTTATCCCTCACAAGTGCCTCAAAAGGAATAAAATTCTTTAAAAGTGCCTAAACGTGTCACAATCCCTTTTTCAATTGCAGCTATCTCGTATCATAAATTGTTTTATATTTAATGCGATCATGTGTTTTCTCTTTTCTGCTAGCTCCTGAGATCTTGAAGTTCGTTGTGAAGATCGATTTGCTCTACTTCTTGTTGCC

> AT1G12730 (251 bp) *AluI* site

GACTTAACGACGCGTATAATGTGGCCTCGTCAACGCAAAGTTCATGTTTTATTTGTTAGTCAATTGTAAGTTGATGTTTTAAATAGCTTCCATCTAAATTGACATATTGATTGGCCATTTTTTGAAAGTTGGGGGATTTAACGACATTTTTCCCTTCATATTTATTAGTTACGGGCCCTAAGCCCGTACCCATTCATAGCCCAACAATCTATTTTAGGATACAGAAATGACAAGTTTCGCTAATTCACACA

>IGN5 (529 bp) No *AluI* site

tcccgagaagagtagaacaaatgctaaaatgtatcatgcggcccaataaccaacaaaactagttgaaagatggatcaatggtttttacatgaagaaagcccaaaccatacactaataatctaatattctattaaaaggacttgctgttattcggcccaatagccaacaaaactaattgaaagatggatcaggggctatggaatacctcagaataaaaagtgttattcattgcagaggaccccttaagcggacatggttgggtccttgttcgacaagacttagtcctccatcttggcctcaagagtgctcgacgaagtttatcaccgcttcatgcggaattttattccttgctatgggcaatagaatgcatgatctctattagcgatacttctggcgcttttgcttccgactgttcagacctgatttctatcattaacaatcaagaagattggccgacattcgcagcggaattgacatcctatcgctccttagtttatttttctccttcttttcgtattagatttcttcc

> AT4G08310 (260 bp) *HaeIII* site

CAAACCCGAACCCAAAACTAAAAATACCCGAATATTTTAATTGAAAATCTAAATTTTACCTAAAACCTGAAATTTTACTCAAAAATCCGGATATTTACCCGAAGTTCCAAAATAGGGCCCAAAAATCTATACCCAAACTAATCCGAACCCGATTAGATGCGAACCGAAATCGAACCGATATTTTAGATTTATCCACCTGGGTTCTAAACTTTTCTACCCGAAAAATCTGTAACCGATCGGGTTTTAACCGAACCCGACCC

>AT5G19257 (254 bp) *HaeIII* site

TTCAAAGATGGAGTTTCACGTGTCGCTCACTACATTTTCTTATTATTATATTCCATATAAAGAGAAACCGAGTAGGGAAAAAACCCTACAAAACCGTGGTTTATTTTTTATTTGGCCGTTTAATACCTGTAGTTTTAAAATTGGAATAAAAATACCAAAATTATTTTTTGTTGCCTTTTAAATTCATGGTCTTTTACATTTTGGTAATTTCGAACTTGCGATTTAACATCTGTTAATGGGTGTTACGGAAGTCG

> AT2G01735 (219 bp) *HaeIII* site

CAAATCTGAAGTCGAACCCAAAAATACATGAATATTTTAATCAAAAATTTAAATTTTATTCGAAATCCGAAAAATTTGCATATTTACCCGCAAAATCAAGTATGGTTCTAAAAAAGGCCTAAAATCTATACACGAACCGATATTTTAGATTTACCCTATAGAATCCTAAACTTCTCTACCCGAAAAACCGTATTCGATCGAATTTTACCCGAATCCGAC

> AT5G27860 (407 bp) No *HaeIII* site

TTCAACCTTTGTTGTTTATTTGTCCACTGGATCTCATATTATTAATACTCCATGCTATTTAACAAAGTCACATATAATATTTGTCTAACAAAACCCCATGAAATATGTAAATATTTATCATGTTATCCAATAAAAACCCATGTAATTTTGTAACAATTCTATATATTTGCTAATATTTCGTGTTTTATAAGAGAATTGCATGCATTTTATAAATATATCATACTATTTCAGAAAAACACATTATTTTAGAAAAATATATATATAGTGTATATATATCGTTTATGTATTTAAAAGAATTACATAATTTTTGTAAATATATCATGCTATTTTAAACAAATACATATAAACTTTATAACTAAAGCTAATGCTATTTTCTGACACAAGATCATTCTCTCAATTCTTTTCCA

> AT3G30380 (319 bp) *DdeI* site

GAGGGAAAGATGATCCGTCAATATAAATAACGTTTAGCAGCCTAAATTTATCGAAAAAGAGACTTGGTGGTTGTGATGGTGGAAGATGTGGTGGTTGTGGTGGTGGAAGAAAGAGAGATGAGTGGTGGTTTGTTCGGATTCATCAAAAGTTTTTCGCCATATAATCATCTAAGTGTTCTGTGTGATTTTGTAAAATAGCATGTAATACATATAAAATTCGACAATGGGTATAAAAGTAATTGCAAATGATGAAAGAGAGTGTATTCTGACACCATAGTTACTTTGTTAATTCTTTTACAATTCATAGTCATTTTGTGCA

> AT1G49490 (272 bp) *DdeI* site

AACCATGTCTCTGCATATTCAATCTAAACATATTCAACTTGATATTCTTGGAAAGTTTAGTTAAGTTCTTGTTATAATCTTATATTTTACGTATTAGTTATAAGGATAGCTCTTCAAACATTTTTCAAATTACTTGTTTTCTTAGAATAAATCTCGAAAAGCTTATATATATTTAATCTGCATATTCATTTAGTTTTTTTCATAATAATCGTCTAATTTTTAGGCAAGAAATATGTGTAACATGTTTGGATTTTATCATCATTTTAGCCTCA

> AT1G35160 (300 bp) *DdeI* site

TTGCTCAATCAACAGAAATTACAAAATGCCAATATGGACGTAAAAGGTCCATTAAGGAATCAAAATACAACAAAGTAACTTTTTCCACATAAAAATAAACTAAGGGCATCTCCAACGCACAGATACAGTATGAGTTTCTCAATTCTATTTTTTTAATCAGTTAATAATAAATATATTATTTATTTAAATTTTATATTATAAAGACAAAATCTGACCAATCATAAATAACAAGGGGTTGAGTTGGGTTTCTCATAGTTTCTCATATGAGAAACGTTTCTCTTTCTTTCTCCACCTTTTTCA

> AT2G36490 (54 bp) No *DdeI* site

GGAAACATGTCCAGCGCTTTAGTTGCACTTACTGCTGAAACTGCTTCTCTTCCA

**Table S3. lncRNAs targeted *MdMYB1* in apple**

| lncRNA ID | lncRNA Gene ID | Status |
| --- | --- | --- |
| TCONS 00024403 | MD03G1044800 | Novel lncRNA |
| TCONS 00056355 | MD06G1096200 | Novel lncRNA |
| TCONS 00079230 | MD09G1257800 | Novel lncRNA |
| TCONS 00101494 | XLOC 069522 | Novel lncRNA |
| TCONS 00024208 | MD03G1030700 | Novel lncRNA |
| TCONS 00105221 | MD12G1099500 | Novel lncRNA |
| TCONS 00041017 | XLOC 028311 | Novel lncRNA |
| TCONS 00022689 | XLOC 015923 | Novel lncRNA |
| TCONS 00063020 | MD07G1019200 | Novel lncRNA |
| TCONS 00058953 | MD07G1058600 | Novel lncRNA |
| TCONS 00122726 | XLOC 084599 | Novel lncRNA |
| TCONS 00142876 | MD16G1145400 | Novel lncRNA |
| TCONS 00053945 | XLOC 037002 | Novel lncRNA |
| TCONS 00085329 | MD10G1071900 | Novel lncRNA |
| TCONS 00140775 | XLOC 097125 | Novel lncRNA |
| TCONS 00049168 | MD05G1208700 | Novel lncRNA |
| TCONS 00008414 | XLOC 006578 | Novel lncRNA |
| TCONS 00119544 | XLOC 082170 | Novel lncRNA |
| TCONS 00026430 | MD03G1182600 | Novel lncRNA |
| TCONS 00028279 | MD03G1015800 | Novel lncRNA |
| TCONS 00036688 | XLOC 025287 | Novel lncRNA |
| TCONS 00105746 | XLOC 072541 | Novel lncRNA |
| TCONS 00050025 | XLOC 034290 | Novel lncRNA |
| TCONS 00050024 | XLOC 034290 | Novel lncRNA |
| TCONS 00121710 | XLOC 083833 | Novel lncRNA |
| TCONS 00139035 | XLOC 095910 | Novel lncRNA |
| TCONS 00048596 | MD05G1168300 | Novel lncRNA |
| TCONS 00053341 | XLOC 036588 | Novel lncRNA |
| TCONS 00045700 | MD05G1005200 | Novel lncRNA |
| TCONS 00054112 | XLOC 037130 | Novel lncRNA |
| TCONS 00074047 | MD08G1171700 | Novel lncRNA |
| TCONS 00038943 | MD04G1182600 | Novel lncRNA |
| TCONS 00035303 | MD04G1188100 | Novel lncRNA |
| TCONS 00018870 | MD02G1302500 | Novel lncRNA |
| TCONS 00053868 | MD06G1168100 | Novel lncRNA |
| TCONS 00123343 | XLOC 085049 | Novel lncRNA |
| TCONS 00074843 | XLOC 051091 | Novel lncRNA |
| TCONS 00012473 | MD01G1078100 | Novel lncRNA |
| TCONS 00018477 | MD02G1276000 | Novel lncRNA |
| TCONS 00079547 | XLOC 054336 | Novel lncRNA |

**Table S4. 24nt siRNA sequences.**

| Locus | sequence |
| --- | --- |
| MdMYB1 | CGUGCAUGUCUGAUAUCCACAGAA |
| MdFLS | UUUGUGUAUCUGAUAUUAUGGUCA |
| MdGST | AAUAAAUGUUUGUCGCAUGAUAUC |
